# *Arundo smaragdina* (Poaceae): a novel species revealed by integrative taxonomy and its implications for the phylogeny of the genus

**DOI:** 10.3389/fpls.2025.1660442

**Published:** 2025-11-17

**Authors:** Fupeng Liu, Boyu Li, Qingmeng He, Aixuan Zhao, Ben Xi, Xiaotong Shen

**Affiliations:** The Energy Plant Research and Development Center, Wuhan Rundo Biotechnology LLC., Wuhan, China

**Keywords:** *Arundo smaragdina*, morphological differentiation, MNP markers, chloroplast genome, transcriptome data

## Abstract

**Introduction:**

*Arundo* species have long served as vital raw materials for human livelihoods, yet their phylogenetic relationships remained poorly resolved until recent decades.

**Methods:**

This study identifies a novel species, *Arundo smaragdina*, through integrative analyses of morphology, multiple nucleotide polymorphism (MNP) markers, and chloroplast genome data, and elucidates its phylogenetic placement within the genus.

**Results:**

*A. smaragdina* is characterized by 72 chromosomes (2n=72), solid and limited rhizomes, erect and branched culms, glabrous nodes, and inflorescences emerging in early September. Mature inflorescences contain 2–5 florets per spikelet, with lemma hairs perpendicularly inserted at the basal region, and the pollen germination rate averages 12.7%. Within the genus, *A. formosana* is confirmed as the basal species. *A. donax* (a species potentially of Asian origin) differs from *A. smaragdina* in having spreading rhizomes and lemma hairs obliquely distributed on the lower quarter, although both species share morphological convergence and similar yields. While *A. smaragdina* is distinct from the Mediterranean *A. plinii* complex (including *A. plinii*, *A. donaciformi*s, and *A. micrantha*), which possesses hollow rhizomes and 1(2) florets per spikelet, it shares similar pollen germination rates and chromosome numbers with some clones of *A. plinii*, and exhibits parallels with *A. micrantha* in yield, chromosome count, and branched culm architecture. At the molecular level, MNP markers confirm the genomic distinctiveness of *A. smaragdina* from *A. donax*, while chloroplast phylogeny reveals its intermediate phylogenetic position between *A. donax* and the *A. plinii* complex. Molecular dating estimates divergence times of approximately 2.29 million years ago (Mya) from *A. plinii* and ~2.9 Mya from *A. donax*.

**Discussion:**

The congruent morphological and molecular evidence suggests that *A. smaragdina* may have played a pivotal role in the evolution of *Arundo* species.

## 1 Introduction

Species identification plays a crucial role in biological research, biodiversity conservation, and species utilization (Wagensommer, 2023; Perrino et al., 2024). Traditional morphological identification methods, still largely used (Di Pietro et al., 2016; Masoumi et al., 2025), while foundational, present limitations as they require extensive taxonomic expertise, access to holotype specimens, and continuous updates to taxonomic keys (Balakrishnan, 2005). With the development of DNA sequencing techniques, DNA markers and chloroplast genome sequencing have been recognized as powerful tools for species discrimination and discovery (Hebert et al., 2003; Antil et al., 2023; Dobrogojski et al., 2020).

Chloroplast genomes, which are maternally inherited in most plants, serve as another valuable tool for species identification, phylogenetics, and evolution studies (Ahmad et al., 2023; Dobrogojski et al., 2020). Chloroplast DNA typically constitutes 5-10% of total cellular DNA extracts (Ahmed, 2015) and has been shown to be fully transcribed (Shi et al., 2016). Multiple tools are currently available for chloroplast genome assembly (Freudenthal et al., 2020), and some studies have successfully reconstructed near-complete chloroplast genomes directly from leaf transcriptome data (Osuna-Mascaró et al., 2018; Senthilkumar et al., 2021). These advancements could significantly improve the resolution of phylogenetic analyses.

*Arundo* belongs to the subfamily Arundinoideae (Teisher et al., 2017). Molecular dating analyses suggest that *Arundo formosana* Hack. is the basal species within the genus (Jike et al., 2020). A recent study by Luo et al. (2024) proposes that *A. formosana* may have diverged from the other *Arundo* species, and *A. donax* L. cv. Lvzhou No. 1, collected from Fujian Province, China, may represent an ecotype with a different genetic origin from the other *A. donax* varieties. Notably, genetic evidence demonstrates that *A. donax* likely originated in Asia and subsequently dispersed globally via human migration (Mariani et al., 2010; Hardion et al., 2014; Canavan et al., 2017). In the Chinese mainland, we inferred the existence of three groups (common, emerald and versicolor) of *A. donax* (Ren et al., 2023a, 2024). But cytogenetic analyses revealed 118 chromosomes in the common group and the versicolor group, whereas the emerald group possesses 72 chromosomes. Whole-genome sequencing estimated a divergence time of 25.03 million years (MYA) between clones 0004 (the common group) and 0408 (the emerald group), and multiple nucleotide polymorphism (MNP) markers detected over 90% polymorphic loci (the percentage of different loci) between the two groups (Ren et al., 2023a, 2023b). These findings suggest that the emerald group likely represents a distinct species within *Arundo*, rather than belonging to *A. donax*.

However, while these preliminary findings from our group suggested the emerald group was a distinct taxonomic entity, a formal taxonomic treatment integrating comprehensive data was lacking. This study was therefore designed to address this knowledge gap by aiming to: 1) Conduct comparative morphological analyses and MNP marker assessments to characterize the emerald and common groups. 2) Further validate the reliability of the near-complete chloroplast genome assembled from transcriptome data, and verify the currently known chloroplast genomes within the genus *Arundo*. 3) Confirm that the emerald group (e.g., clone 0408) represents a novel species within the genus *Arundo* based on chloroplast genome data, and distinguish its morphological differences from other *Arundo* species.

## 2 Materials and methods

### 2.1 Morphological and cytological trait survey and analysis

A total of 118 *Arundo* clones collected from the Chinese mainland, comprising 49 emerald group clones and 69 common group clones, were subjected to morphological analyses. Morphological analysis was divided into two parts: field observation and laboratory analysis.

Field studies were conducted at the Yueyang Experimental Station (113.01, 29.47; WGS84), Hunan Province, China. Clones were planted in April 2020 in 94 m² plots (1.4 × 1.4 m spacing), with 2 m buffer zones between plots. During autumn 2023, morphometric assessments were performed. For each clone, 11 traits were quantified in 10 randomly selected individuals. These traits were selected from a prior investigation (data not shown) of 31 traits across 9 clones due to their suitability for large-scale field measurement and their observed variation between the two groups. The selected traits including: culm height (CH), number of culm internodes per culm (NOCIPC), middle ten culm internodes length (excluding inflorescence) (MTCIL), basal culm stem internode diameter (BCSID), basal culm internode wall thickness (BCIWT), leaf length of the sixth leaf from top (LLOTSLFT), leaf width of the sixth leaf from top (LWOTSLFT), aspect ratio of leaf (AROL), inflorescence width (IW), inflorescence length (excluding peduncle) (ILEP), aspect ratio of inflorescence (AROI). PCA (using ade4) and box-and-whisker plots (generated via ggplot2) were applied to analyze morphological traits and detect potential clusters among clones.

Laboratory analyses were conducted for the inflorescence structure, rhizome growth patterns, and chromosome number of the common and emerald groups. Pollen viability in the common and emerald groups was assessed via I_2_-KI staining: normal, fertile pollen grains exhibited dark blue staining, whereas abnormal pollen grains remained unstained. Following the method of Hardion et al. (2015), we additionally performed pollen germination experiments.

### 2.2 MNP marker analysis

Leaf samples were collected from 118 clones, and genotyped using 1,100 MNP markers developed by Ren et al. (2023a). Following the method of Liu J. et al. (2024), markers with 100% amplification success across all clones were selected. Amplified sequences of clones by same maker were aligned using MAFFT v7.526 (Katoh and Standley, 2013) with default parameters. Concatenated sequences, generated by Concatenator v0.3.1 (Vences et al., 2022), served as input for phylogenetic reconstruction in IQ-TREE v2.4.0 (Minh et al., 2020). The best-fit model (GTR +F+R4) was determined by IQ-TREE according to the Bayesian Information Criterion (BIC), and 1,000 ultrafast bootstrap replicates were performed to assess node support. The final tree was visualized using iTOL (Letunic and Bork, 2024). Subsequently, genetic similarity (GS) among clones from the two groups was assessed using the formula proposed by Liu J. et al. (2024), and visualized as heatmaps via ggplot2.

### 2.3 Chloroplast genome assembly and comparative analysis

For chloroplast genome assembly, transcriptome data from BioProject PRJEB36611 (Jike et al., 2020) were used to assemble near-complete chloroplast genomes for five *Arundo* species (*Arundo plinii* Turra; *Arundo donaciformis* (Loisel.) Hardion, Verlaque & B. Vila; *Arundo micrantha* Lam.; *Arundo formosana* Hack.; *Arundo donax* L.) and three outgroup species (*Molinia caerulea* L.; *Hakonechloa macra* Makino; *Phragmites australis* (Cav.) Trin. ex Steud). Whole-genome sequencing and transcriptome data for clones 0004 and 0408, obtained from our previous studies (Ren et al., 2023b, 2024), were utilized to assemble complete and near-complete chloroplast genomes for these two clones. BioProject PRJNA974205 (Luo et al., 2024) provided data for the reassembly of the complete chloroplast genome of *A. donax* cv. Lvzhou No.1.

Additionally, complete chloroplast genomes of three *Arundo* species—*A. formosana* (NC_054211.1, MZ620725.1), *A. plinii* (NC_034652.1), and *A. donax* (NC_037077.1)—along with three outgroup species (*M. caerulea* NC_033980.1, *H. macra* NC_025235.1, *P. australis* NC_022958.1) from subfamily Arundinoideae (Poaceae), were retrieved from NCBI GenBank to infer interspecific evolutionary relationships. The boundary of *A. formosana* (NC_054211.1) follows the description by Feng et al. (2021).

Complete chloroplast genomes were assembled using GetOrganelle v1.7.7.0 (Jin et al., 2020) with default parameters. Near-complete chloroplast genomes were assembled following the methodology of Osuna-Mascaró et al. (2018), with *A. donax* (NC_037077.1) as reference. The starting positions of the assembled chloroplast genomes were standardized to align with the reference genome of *A. donax* (NC_037077.1). Chloroplast genome annotation was performed using CPGAVAS2 (Shi et al., 2019), while tRNA detection employed tRNAscan-SE v1.21 (Schattner et al., 2005). The circular chloroplast genome map was subsequently generated using CPGView (Liu et al., 2023). Chloroplast genome comparisons were performed using mVISTA (LAGAN algorithm mode) with *A. donax* (NC_037077.1) as the reference genome (Frazer et al., 2004). Sequence similarity between *A. donax* and other *Arundo* species with complete chloroplast genomes was further assessed via Circoletto (Darzentas, 2010) under an E-value threshold of 1×10⁻^10^. Genome junction sites were visualized using CPJSdraw (Li et al., 2023).

### 2.4 Phylogenetic analysis and divergence time estimation

All assembled chloroplast genomes were analyzed to resolve the evolutionary relationships within *Arundo*. Sequences were aligned with MAFFT v7.526 (Katoh and Standley, 2013), trimmed using TrimAL v1.4 (Capella-Gutiérrez et al., 2009), and used to reconstruct a maximum-likelihood tree in IQ-TREE v2.4.0 (Minh et al., 2020), with the best-fit model (GTR+F+I+G4) determined by IQ-TREE according to the BIC and 1,000 ultrafast bootstrap replicates performed to assess node support. The final tree was visualized using iTOL (Letunic and Bork, 2024).

Divergence time estimation included four complete *Arundo* chloroplast genomes (*A. plinii*, clone 0004, clone 0408 and *A. donax*) with *P. australis* as the outgroup. We employed the *Phragmites*-*Arundo* divergence time of 29.0 Mya (95% CI: 19.6-38.3) proposed by Christin et al. (2013), which was validated by Hardion et al. (2015) in *A. formosana* ecotypes. This molecular dating exhibited temporal concordance with the orogenesis of the center mountain range (~2 Ma). The divergence time estimates were performed using the MCMCTree v4.9e program (Yang, 2007).

## 3 Results

### 3.1 Morphological and cytological differentiation across clones

**Figure 1:**
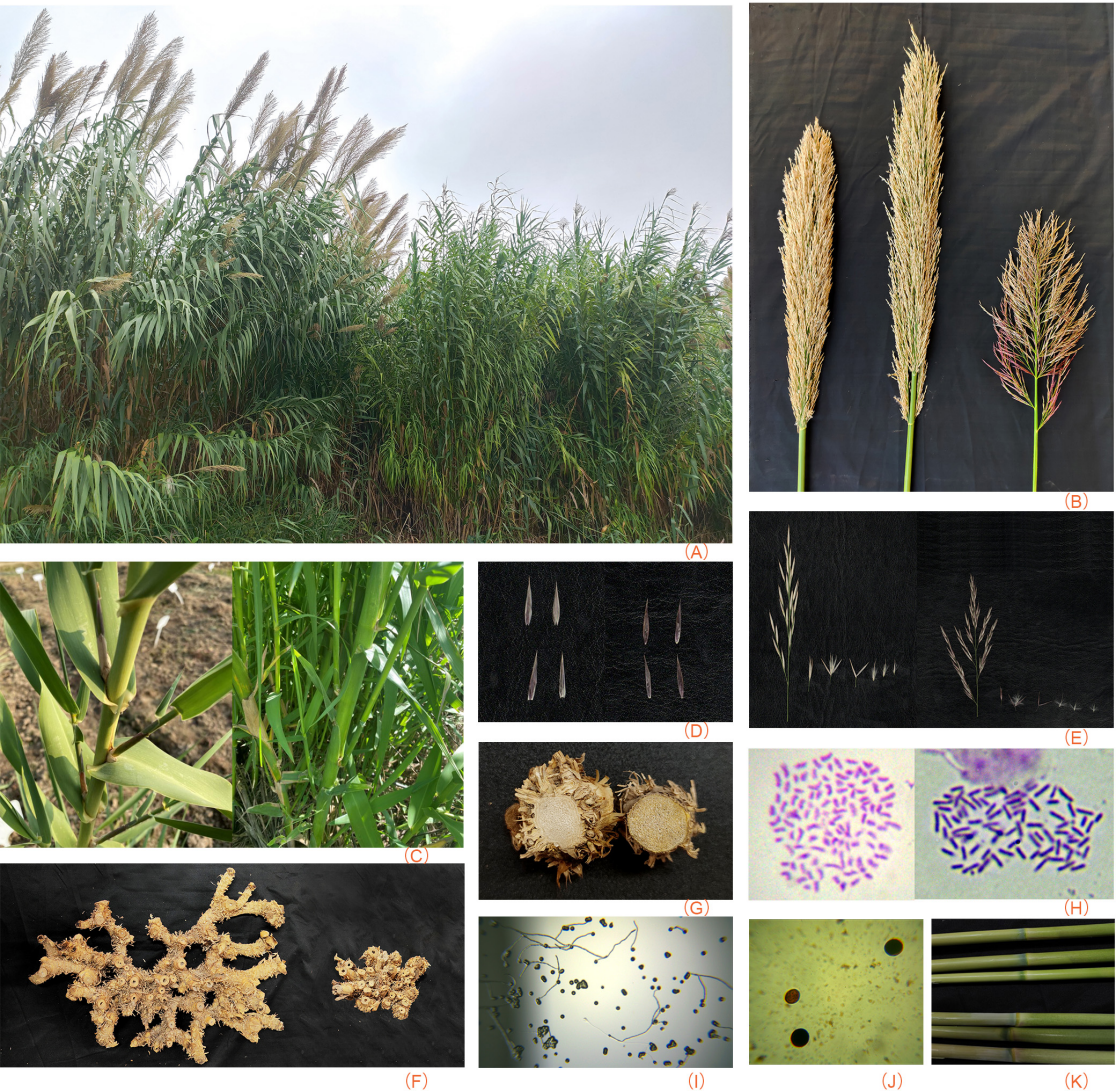
Comparison morphological and cytological traits between the common and the emerald group. **(A)** Plants after 2 years (photographed in mid-September 2024): Common (left), emerald group (right). **(B)** Fully emerged inflorescence: Common (left two), emerald group (right). **(C)** Branched culms: Common (left), emerald group (right). **(D)** Glumes (top: lower glumes [exterior and interior], bottom: upper glumes [exterior and interior]): Common (left), emerald group (right). **(E)** Inflorescence structures (from left to right: secondary branch, spikelet, expanded spikelet, glumes, three florets): Common (left), emerald group (right). **(F)** Rhizomes after 1 year: Common (left), emerald group (right). **(G)** Rhizome cross-sections: Common (left), emerald group (right). **(H)** Chromosomes (100×): Common (left), emerald group (right). **(I)** Pollen germination of the emerald group. **(J)** Pollen staining of the emerald group. **(K)** Glabrous node (top three: common group, bottom three: emerald group).

Field observations comparing the emerald and common groups identified significant divergence in morphological and phenological traits. The emerald group exhibited a conspicuously brighter green leaf coloration, which emerged as the most diagnostic feature enabling effortless visual distinction between the two groups in field conditions. In terms of flowering period, the common group initiated flowering approximately one and a half months earlier than the emerald group (**Figure 1A**). The inflorescence of the emerald group was shorter than that of the common group (**Figure 1B**; **Supplementary Table S1**), but both had branched culms (**Figure 1C**). A distinct contrast was observed in rhizome growth patterns (**Figure 1F**). Unlike the common group whose rhizomes grew upwards in a spreading pattern, the emerald group displayed limited rhizome growth without upward tendencies, though both groups had solid (non-hollow) rhizomes (**Figure 1G**).

Laboratory observations focused on reproductive structures provided additional insights into the divergence between the two groups. Both groups bore 2–5 florets per spikelet. However, significant differences were noted in the lemma hair distribution and orientation. The lemma hair of the emerald group was primarily distributed in the middle to lower regions of the lemma and exhibited a non-erect orientation, in contrast to the common group (**Figure 1E**). No discernible differences were observed in the glumes between the two groups (**Figure 1D**).

In terms of chromosome number, the emerald group had a lower chromosome count (2n = 72) compared to the common group (2n = 108; **Figure 1H**). Pollen viability assays further differentiated the two groups. Clones of the common group predominantly produced small, irregularly shaped pollen grains. In contrast, the emerald group clones (e.g., clone 0408) exhibited partial pollen staining and higher pollen germination (approximately 12.7%; **Figures 1I, J**).

To further investigate the differences between the two groups, PCA was performed on 11 morphological traits, revealing that the 118 clones were grouped into two phenotypically divergent groups (**Supplementary Figure S1**; **Supplementary Table S2**). Box-and-whisker plots analysis of traits further revealed significant distribution variations among the two groups, with substantial overlapping observed in most traits (**Supplementary Figure S2**). In terms of culm height, the common group was higher than the emerald group, which was consistent with the finding that the length of the middle ten internodes in the common group was greater than that in the emerald group. Interestingly, the emerald group had a larger number of internodes compared to the common group. Notably, no obvious differences were found in the basal stem thickness between the two groups.

Differences in flowering periods, lemma hair distributions, and pollen viability suggest potential reproductive isolation between the two groups. The PCA of 11 traits and significant rhizome morphological divergence indicate adaptation to distinct environmental conditions. These results clearly distinguish the emerald group from the common group.

### 3.2 Phylogenomic relationships via MNP markers

**Figure 2:**
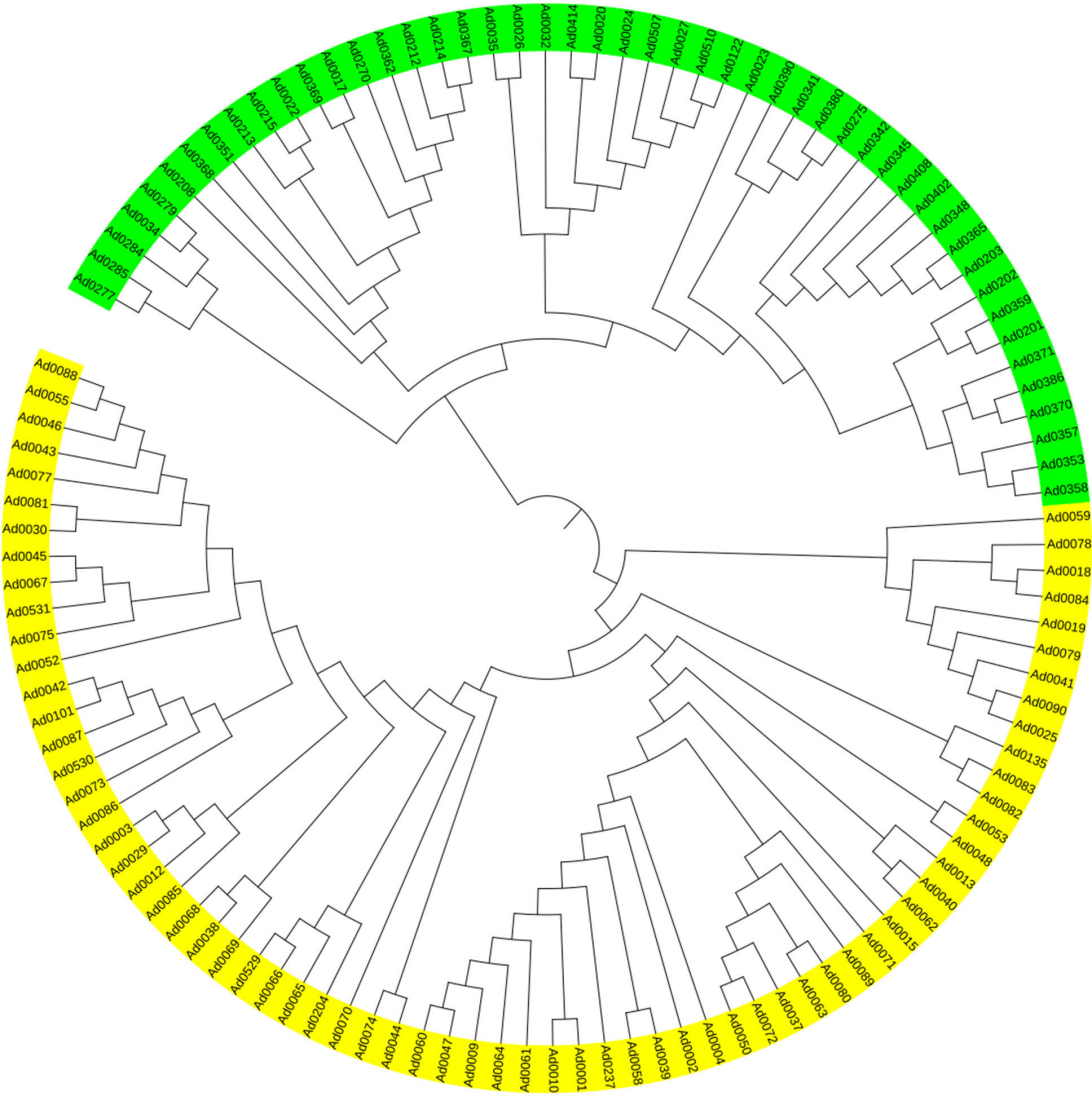
Clustering dendrogram based on MNP markers for 118 clones. Yellow and green represent the common group and the emerald group, respectively.

To further confirm that the emerald group does not belong to the common group at the genomic level, MNP markers were employed to genotype the two groups. The number of shared MNP markers among different clones ranged from 495 to 947 (**Supplementary Table S3**). A total of 304 shared MNP markers were used to classified 118 clones into two distinct groups: Clade I consisted exclusively of clones from the common group, whereas Clade II was composed solely of emerald group clones (**Figure 2**). These results indicate that the emerald group is genomically distinct from the common group at the genome level.

**Figure 3:**
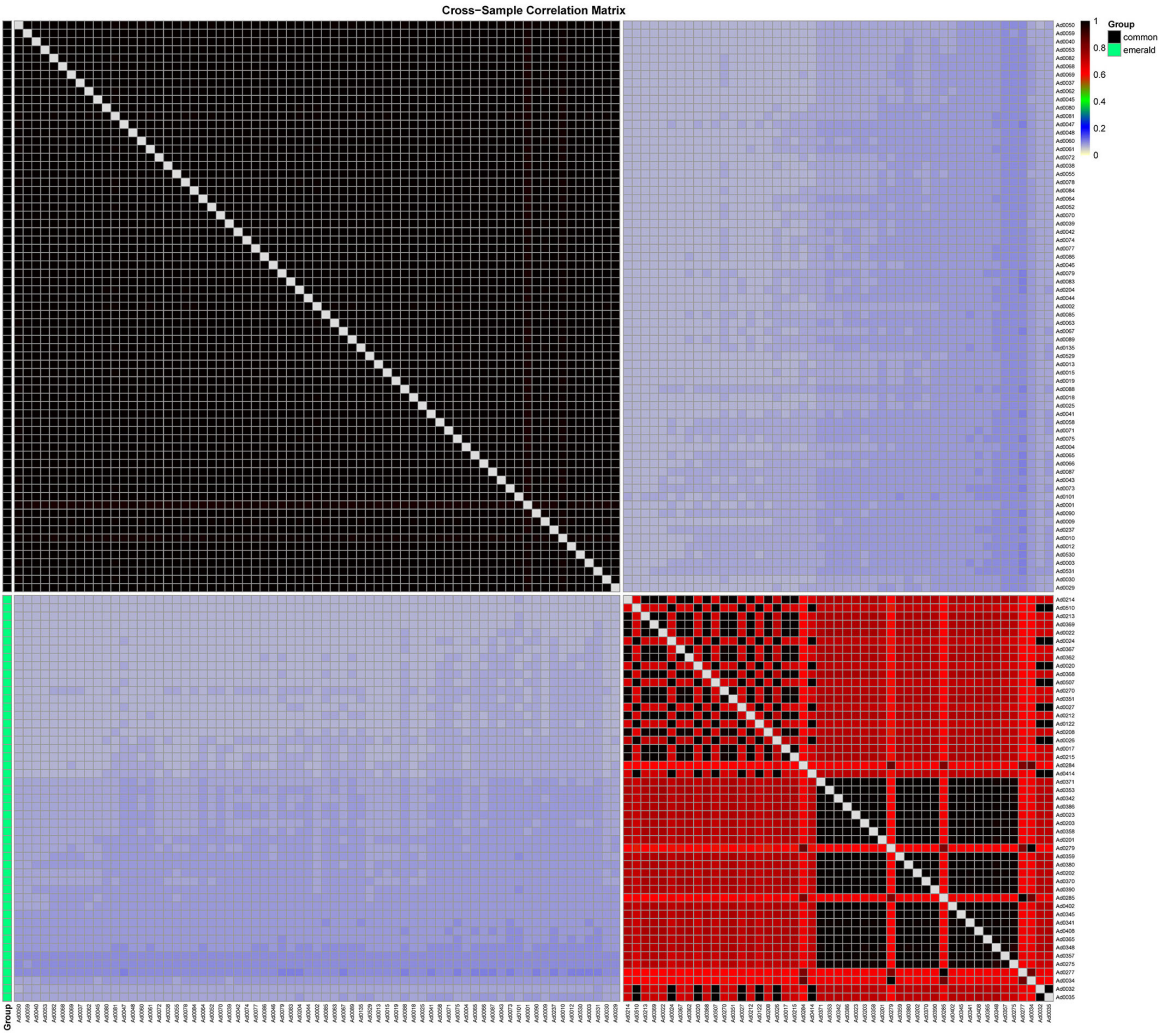
Heatmap of genetic similarity between two groups. The common and emerald groups are color-coded in black and green, respectively, on the left side. The genetic similarity values are provided in the top-right corner.

Based on genetic similarity, the emerald group was also be distinguished from the common group. The genetic similarity between the common group and the emerald group was only 6.05%-10.32%, demonstrating a profound genomic divergence consistent with species-level separation (**Figure 3**). Within-group genetic similarity was markedly higher in the common group (96.2%-100%; **Supplementary Figure S3**) than in the emerald group (58.9%-100%; **Supplementary Figure S4**), indicating that the emerald group possesses a more diverse gene pool. This pattern aligns with morphological observations, where the common group exhibited significantly lower pollen viability compared to the emerald group (**Figure 1I**). These findings collectively demonstrate that the emerald group represents a genomically distinct entity from the common group (*A. donax*). This genomic differentiation aligns with morphological and cytological observations, suggesting the emerald group represents a novel taxon in the Chinese mainland.

### 3.3 Chloroplast genome assembly

To further confirm that the emerald group does not belong to *A. donax* or the other four species within the genus *Arundo*, further research was conducted at the chloroplast genome level. Based on NGS sequencing data, the complete chloroplast genomes of clones 0408 and 0004 were successfully assembled. Additionally, the complete chloroplast genome of *A. donax* cv. Lvzhou No. 1 was also successfully reassembled from its raw data. Assembly of transcriptome data yielded 12 near-complete chloroplast genomes. All assembled chloroplast genomes are listed in **Supplementary Table S4**.

The complete chloroplast genome of clone 0408 (137,185 bp) exhibited a typical quadripartite angiosperm structure, comprising large single-copy (LSC: 82,092 bp), small single-copy (SSC: 12,613 bp), and inverted repeat (IR: 21,240 bp) regions (**Supplementary Figure S5**). The complete chloroplast genome of clone 0004 was identical to that of *A. donax* cv. Lvzhou No. 1 as reassembled (**Supplementary Figure S6**). Published *A. formosana* genomes (MZ620725.1, NC_054211.1) are completely identical (**Supplementary Table S5**).

### 3.4 Chloroplast genome comparison

Compared with the complete chloroplast genomes of clones 0004 and 0408, the near-complete chloroplast genomes exhibited significantly shorter lengths across four regions. Notably, all nine near-complete chloroplast genomes of *Arundo* species shared identical low-similarity and missing regions, including extensive sequence deletions near 84–87 k bp, 92–93 and 132–134 k bp (**Supplementary File S1**). Furthermore, sequencing strategy significantly influenced genome assembly quality. For example, the near-complete chloroplast genome assembled from BioProject PRJEB36611 exhibited deletions in the 46–48 and 64–65 k bp regions, whereas clones 0004 and 0408 displayed no such gaps, highlighting the impact of sequencing methodology on sequence integrity and confirming the feasibility of assembling a complete chloroplast genome from transcriptome data.

**Figure 4:**
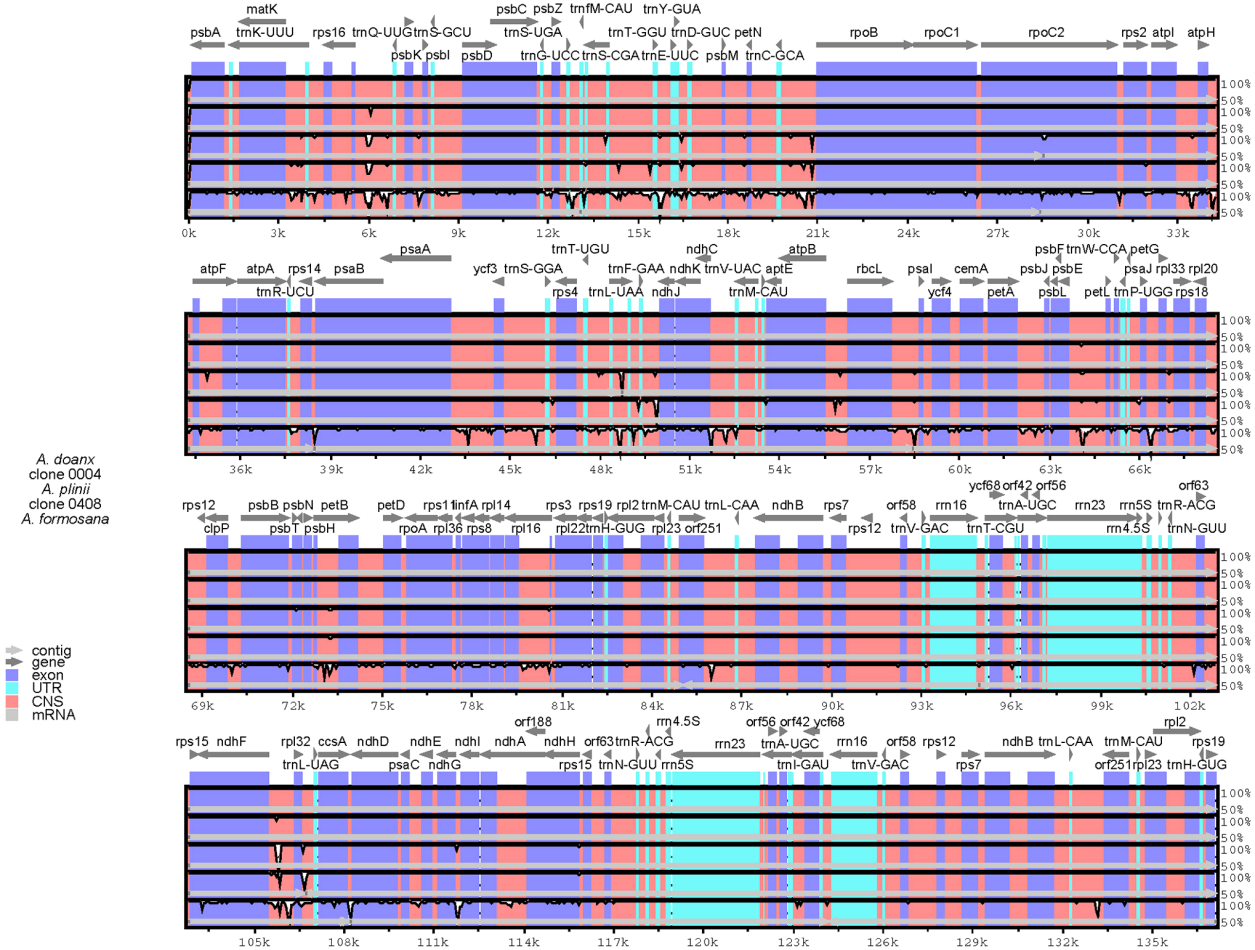
Sequence variation plots among the *Arundo* chloroplast genomes. Annotated genes are displayed on the top. The vertical scale represents the percentage within 50–100% homogeneity. The color legend is summarized in the lower left-hand corner.

Using *A. donax* (NC_037077.1) as the reference, a comprehensive genomic variation analysis of the complete chloroplast genomes across *Arundo* species was performed (**Figure 4**). Results revealed that all analyzed genomes, except *A. formosana*, exhibited high similarity to *A. donax*. The inclusion of chloroplast genomes assembled from transcriptome data in the comparative analysis similarly yielded consistent results (**Supplementary File S1**), further confirming that the emerald group (e.g., clone 0408) belongs to the genus *Arundo* and demonstrating the reliability of chloroplast genomes assembled from transcriptome data.

Notably, in contrast to the reference genome *A. formosana* (NC_054211.1), the near-complete chloroplast genome of *A. formosana* assembled from transcriptome data showed higher overall similarity to *A. donax* (**Supplementary File S1**). Furthermore, collinearity analysis (**Supplementary Figure S7**) revealed that the reference genome *A. formosana* (NC_054211.1) exhibited lower similarity to *A. donax* than *P. australis* or *M. caerulea*, with most regions showing less than 25% collinearity. The reference genome *A. formosana* (NC_054211.1) may warrant further investigation. In contrast, *A. plinii* showed the best collinearity with *A. donax*, followed by clone 0408, indicating that clone 0408 differed from both *A. plinii* and *A. donax* at the chloroplast genome level.

**Figure 5:**
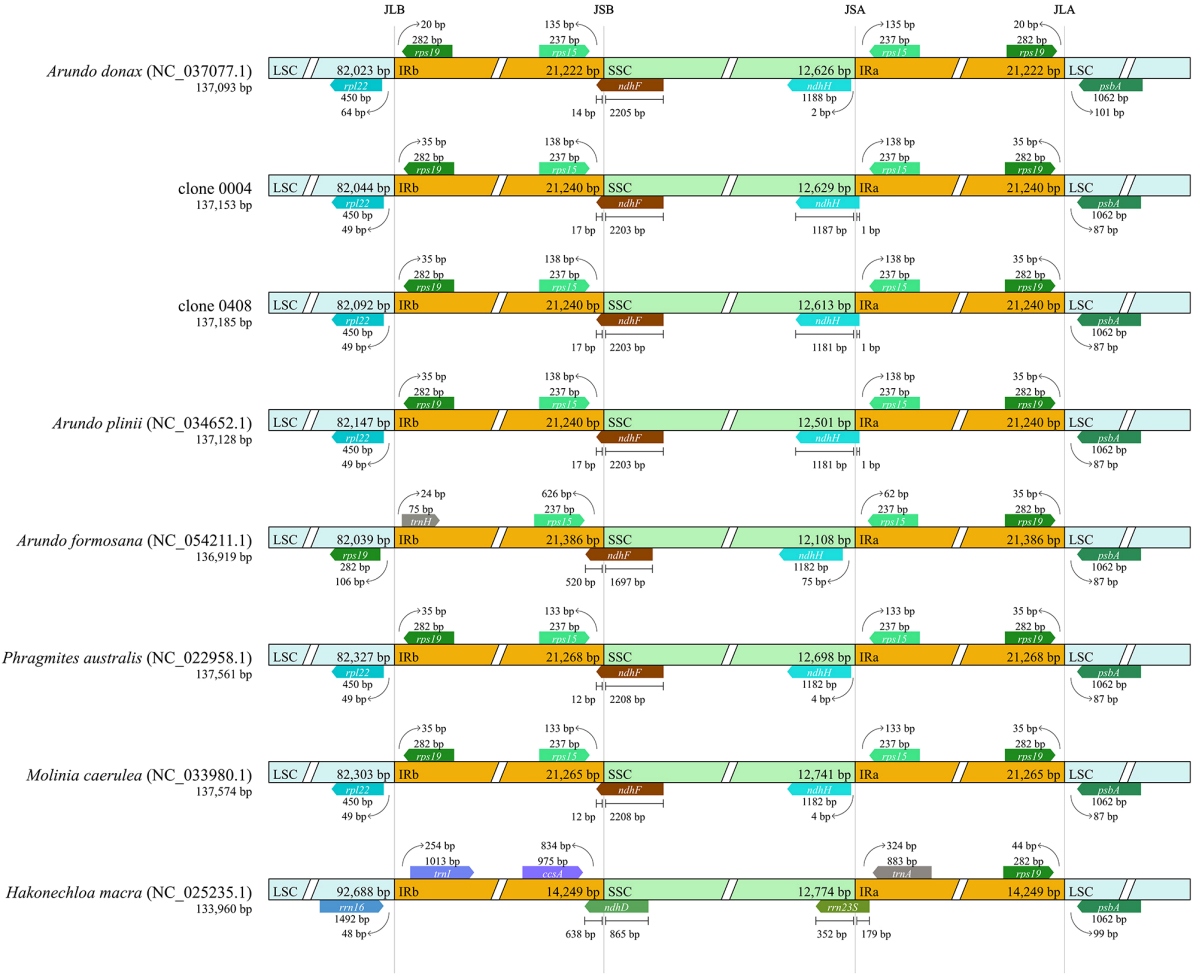
Comparison of inverted repeat (IR) region boundaries in chloroplast genomes.

Inverted repeat (IR) boundary analysis revealed that *A. plinii*, clones 0004 and 0408 within the genus *Arundo* shared identical IR boundaries and lengths. In contrast, the Italian *A. donax* (NC_037077.1) exhibited an IR contraction compared to clone 0004, a Chinese *A. donax* clone (**Figure 5**; **Supplementary Table S6**). The observed IR contraction in the Italian *A. donax* could reflect an evolutionary change following geographic isolation, though its functional significance requires further study.

The high similarity at the chloroplast genome level further confirmed that the emerald group belongs to the genus *Arundo*. However, collinearity analysis revealed significant differences in the chloroplast genomes between the emerald group (e.g., clone 0408) and other *Arundo* species, providing further evidence that it is likely a new species within the genus.

### 3.5 Phylogenetic resolution of *Arundo*

**Figure 6:**
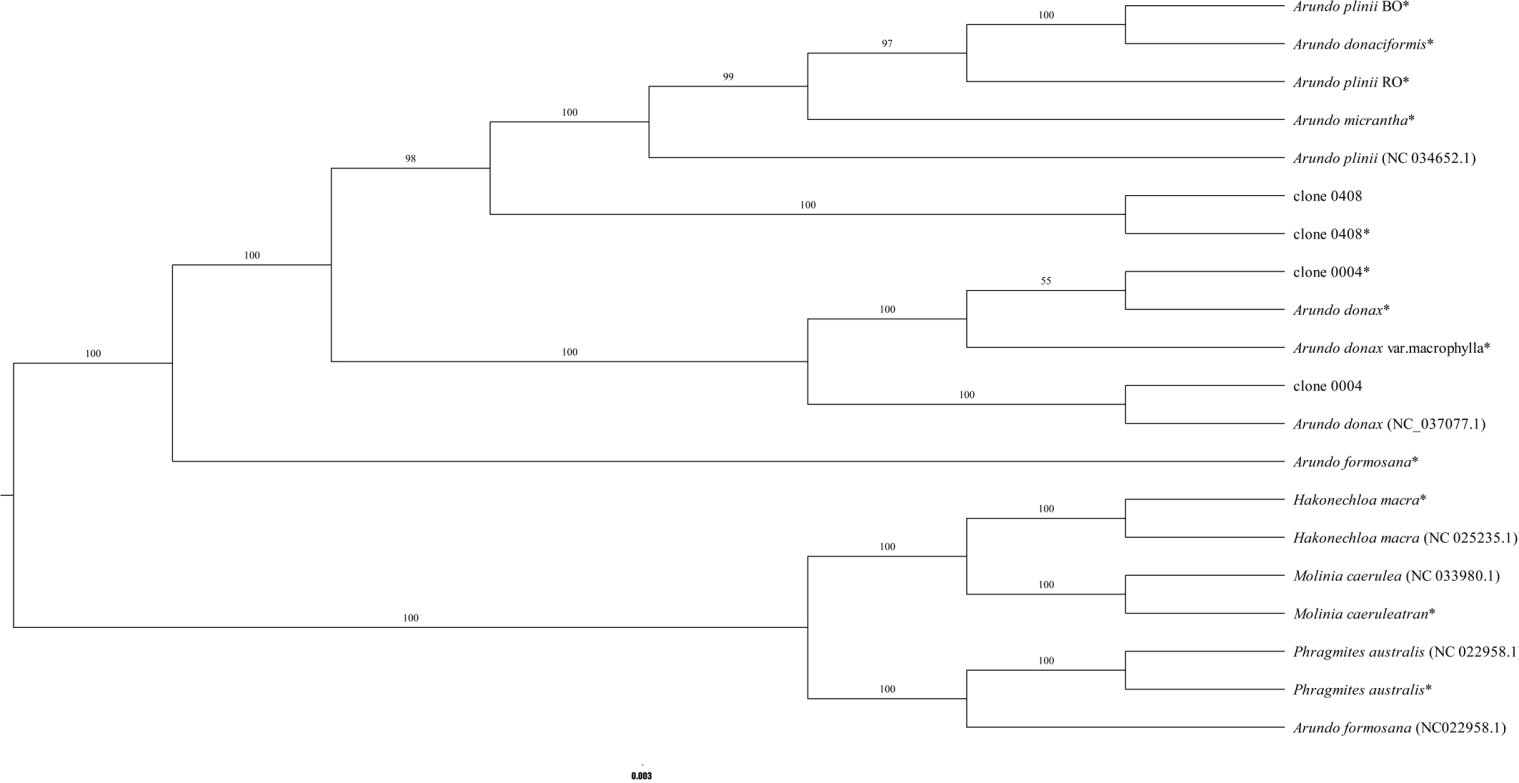
Phylogenetic relationships of *Arundo* species inferred from chloroplast genomes. “*” indicates a nearly complete chloroplast genome assembled from transcriptome data.

To determine the phylogenetic relationships between the emerald group and other species within the genus *Arundo*, a chloroplast genome-based phylogenetic tree was constructed. In the maximum likelihood (ML) phylogenetic tree (**Figure 6**; **Supplementary Figure S8**), the near-complete chloroplast genomes, with the exception of *A. formosana*, clustered with their corresponding complete reference genomes, confirming the reliability of transcriptome-derived chloroplast data.

Clone 0408 did not cluster with any recognized *Arundo* species, forming an independent lineage that robustly supports its designation as a novel species. Clone 0004, whose chloroplast genome sequence is identical to that of *A. donax* cv. Lvzhou No.1, formed a clade with *A. donax* (NC_037077.1). A notable discrepancy was observed in the phylogenetic placement of *A. formosana*. While the near-complete chloroplast genome was clustered within the genus *Arundo* as expected, the published reference chloroplast genome of *A. formosana* (NC_054211.1) formed a distinct clade with *P. australis* (NC_022958.1) and other outgroup species (**Figure 6**; **Supplementary Figure S8**). This anomalous phylogenetic placement, inconsistent with the transcriptome-based assembly, reinforces the concern raised in section 3.4 that the reference genome of *A. formosana* (NC_054211.1) may warrant further investigation.

Chloroplast genome divergence provides further validation that the emerald group (e.g., clone 0408) represents a novel species. Divergence from other species in nuclear and chloroplast genomes, morphological traits, and cytological features indicates adaptive modifications during its historical evolutionary process. We propose the name *Arundo smaragdina* for this species, reflecting its distinctive leaf pigmentation.

### 3.6 Divergence time analysis

**Figure 7:**
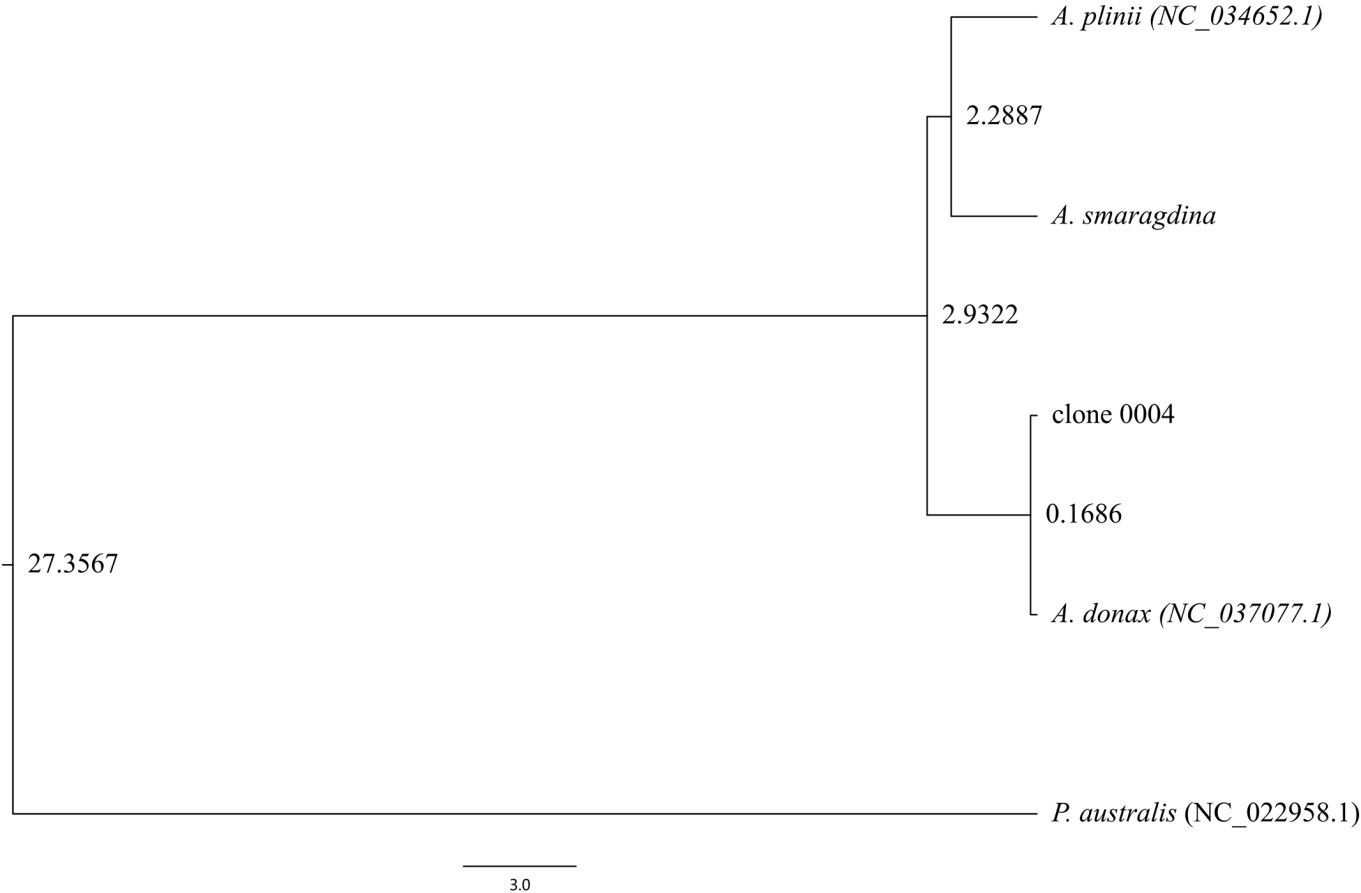
Chronogram of the genus *Arundo*.

Integration of the *A. smaragdina* chloroplast genome with published *Arundo* genomes and the outgroup *P. australis* revealed key divergence events within the genus (**Figure 7**). Phylogenetic analysis indicated that *A. smaragdina* and *A. plinii* diverged 2.29 million years ago (Mya), with this lineage splitting from *A. donax* 2.9 Mya. Within *A. donax*, the Italian clone (NC_037077.1) and clone 0004 diverged more recently, approximately 0.17 Mya.

This temporal evidence, consistent with the morphological, cytological, and genomic divergences documented in previous sections, offers robust and multi-layered confirmation of *A. smaragdina* as a distinct species within the genus *Arundo*.

## 4 Discussion

### 4.1 MNP markers support genomic divergence between *A. smaragdina* and *A. donax*

Previous studies have employed various molecular markers and partial chloroplast genes to explore intraspecific diversity within *A. donax*, but these approaches demonstrated limited discriminatory power for distinguishing *A. donax* clones (Bucci et al., 2013; Pilu et al., 2013; Touchell et al., 2016; Liu et al., 2019; Tarin et al., 2013). To address this, we developed 1,100 MNP markers, which mapped uniformly across the *A. donax* reference genome (Ren et al., 2023a, 2024; **Supplementary Figure S9**). MNP marker technology has emerged as a powerful tool for variety identification, demonstrating success across diverse species including *Oryza sativa* L., *Dendranthema morifolium* (Ramat.) Tzvelev, *Lentinula edodes* (Berk.) Pegler, and *Vitis vinifera* L (Fang et al., 2021; Ling et al., 2023; Liu J. et al., 2024; Liu Y. et al., 2024). Phylogenetic clustering of *A. smaragdina* and *A. donax* into distinct groups underscores their divergence at the genomic level (**Figure 2**).

The significantly lower genetic similarity among different clones of *A. smaragdina*, compared to the high clonal uniformity within *A. donax* (**Supplementary Figures S3**, **S4**; **Supplementary Table S3**), suggests a history of sexual reproduction and the maintenance of a more diverse gene pool, which is consistent with the extensive phenotypic variation (e.g., in leaf morphology, tiller number, and flowering time) observed in *A. smaragdina* in the field. However, *A. donax* exhibits relatively limited phenotypic variation in the field. The most common phenomenon is that some individuals of the common group transform into the versicolor groups due to somatic mutations resulting foliar variegation (Antal et al., 2018; Guarino et al., 2019; Danelli et al., 2020; Ren et al., 2023b). SRAP and TE-based molecular markers failed to detect genetic differentiation between these morphotypes (Ahmad et al., 2008). Comprehensive differences in genetic (MNP markers), cytological (chromosome number), reproductive (pollen germination rate), and ecological (ecotype diversity) traits demonstrate potential early reproductive isolation mechanisms, indicating that *A. smaragdina* and *A. donax* split into distinct species early in their evolutionary history.

### 4.2 Morphological and cytological evidence for *A. smaragdina* as a new species

The genus *Arundo* exhibits substantial morphological plasticity (Ngernsaengsaruay et al., 2023; Cantaluppi et al., 2015). In this genus, *A. smaragdina* exhibits distinct morphological differences compared to the other five species.

Compared to other *Arundo* species, *A. formosana* exhibits a prostrate growth habit, ~1 m height, and is endemic as a pioneer grass in estuarine environments (Hardion et al., 2017a). Its restricted distribution further underscores its taxonomic uniqueness within the genus (Liu and Sylvia, 2006; Lee et al., 2022).

The *A. plinii* complex (*A. plinii*, *A. donaciformis*, and *A. micrantha*) represents a circum-Mediterranean taxon, distinguished from *A. donax* and *A. smaragdina* by its spikelet structure, which bears 1(2) florets compared to 3–5 florets in the latter two (**Figure 1E**; Danin, 2004; Hardion et al., 2012a). Additionally, the *A. plinii* complex exhibits thinner rhizomes characterized by a parenchymous cross-section with a central lumen (Danin and Naenny, 2008), contrasting sharply with the solid, lumenless rhizomes of *A. donax* and *A. smaragdina* (**Figure 1G**).

Among the complex members, *A. micrantha* is further differentiated by a larger culm diameter exceeding 5 mm under the panicle (vs. <4 mm in *A. plinii* and *A. donaciformis*; Danin, 2004; Hardion et al., 2012a); and greater similarity to *A. smaragdina* (4.7-12.9 mm) and *A. donax* (9.3-18.2 mm). Intriguingly, *A. smaragdina* shares rhizome growth patterns with *A. micrantha* (**Figure 1F**; Danin, 2004). However, the inflorescence architecture of *A. micrantha* more closely resembles that of *A. donax*, differ significantly from *A. smaragdina* (**Figure 1B**; Hardion et al., 2012b; Tomàs et al., 2019; Ferrer-Gallego and Hardion, 2025). The branched culms of *A. micrantha* distinguish it very well from *A. plinii* (Danin, 2004); however, *A. smaragdina* and *A. donax* also have branched culms (**Figure 1C**).

*A. donaciformis* exhibits pubescent nodes and hairy upper glumes, while *A. micrantha* and *A. plinii* s.str. are marked by glabrous nodes and glabrous upper glumes, respectively (Hardion et al., 2012a). Moreover, *A. donax* and *A. smaragdina* are glabrous at the nodes, lower glumes, and upper glumes (**Figures 1D, K**). Additionally, in *A. smaragdina*, hairs are primarily distributed in the middle to lower regions of lemma and exhibit a non-erect orientation similar to *A. donaciformis*, whereas in *A. donax*, hairs are notably longer and closely appressed to the lemma surface (**Figure 1E**; Hardion et al., 2012a).

Regarding Italy and Asian *A. donax* (e.g., clone 0004), the two exhibit no significant morphological differences in traits such as the lemma and root system (**Figure 1**; Danin, 2004; Hardion et al., 2012a), indicating the reliability of morphological identification for species differentiation. Furthermore, both this study and the research by Hardion et al. (2012a) demonstrate that PCA and box-and-whisker plots analysis are also important tools for species discrimination within the genus.

Pollen germination and chromosome number also provide valuable insights for species identification. Germination rates of *A. smaragdina* align closely with those of *A. plinii* and *A. donaciformis* within the genus (**Figure 1I**; **Supplementary Table S7**; Hardion et al., 2015). However, unlike *A. donax* populations in the Middle East that exhibit seed set (Danin, 2004; Hardion et al., 2014), no seeds were observed in *A. smaragdina* or *A. donax* in the Chinese mainland under *ex situ* conditions. This sterility in *A. smaragdina* may mirror the reproductive behavior of *A. plinii*, which produces seeds exclusively in its native habitat (Hardion et al., 2015). Cytogenetic analyses further differentiate *A. smaragdina*: its karyotype (2n=72) contrasts with *A. donax* (2n=108) and *A. donaciformis* (2n=108), while aligning with *A. micrantha* (2n=70-72) and some cytotypes of *A. plinii* (2n=72, 74, 76, 108, 114; Hardion et al., 2015).

Collectively, *A. smaragdina* is morphologically distinct from all known *Arundo* species. However, its morphological convergence with *A. donax*—including shared traits such as plant height and perennial habit—likely resulted in its historical misclassification as a non-distinct taxon within the genus.

### 4.3 Chloroplast genome support *A. smaragdina* as a new species

Species-specific clustering validated the utility of transcriptome-derived chloroplast genomes, as these genomes consistently grouped with their complete genome counterparts, consistent with previous studies (**Figure 6**; **Supplementary Figure S8**; Osuna-Mascaró et al., 2018; Senthilkumar et al., 2021). In contrast to studies using partial chloroplast genes for interspecific comparisons, nearly complete chloroplast genomes provide more comprehensive genetic information, particularly when species divergence times are short. For example, Jike et al. (2020) analyzed *Arundo* species using five intergenic regions, yielding results slightly divergent from nuclear genome-based studies (specifically the phylogenetic relationships among three species in the *A. plinii* complex). In contrast, our findings based on nearly complete chloroplast genomes showed strong consistency with nuclear genome analyses (**Figure 6**, **Supplementary Figure S8**; Jike et al., 2020). Furthermore, the near-complete chloroplast genome of *A. smaragdina* clustered with its complete genome, supporting its novel species status.

A comparative analysis of the chloroplast genome between *A. smaragdina* and other *Arundo* species revealed high sequence similarity between *A. smaragdina* and other species, whereas notable differences were observed in the IR regions: *A. smaragdina* exhibited greater similarity to the IR regions of *A. plinii* and the Chinese mainland *A. donax*, while the IR region of Italian *A. donax* was contracted. These results imply that *A. smaragdina* contributed to the evolutionary radiation of *Arundo* species.

### 4.4 Interspecific relationships within genus *Arundo*

Phylogenetic topology aligned with prior studies, positioning *A. formosana* as the basal taxon within the genus *Arundo* (**Figure 6**; **Supplementary Figure S8**; Jike et al., 2020). Interestingly, the published *A. formosana* (NC_054211.1) chloroplast genome did not cluster with the near-complete chloroplast genome, but instead grouped with *P. australis* and other outgroup species, a finding consistent with other phylogenetic studies that also reported its clustering with species such as *Crinipes abyssinicus* Hochst. and *Crinipes longifolius* C.E.Hubb., outside the core *Arundo* lineage (**Supplementary Figure S8**; Luo et al., 2024). The genomic comparisons (**Supplementary File S1**) also consistently demonstrate significant differences between the *A. formosana* reference genome (NC_054211.1) and *A. donax* (NC_037077.1). Furthermore, similarity analysis (**Supplementary Figure S7**) indicates that their degree of similarity is even lower than that between *A. donax* and either *P. australis* or *M. caerulea*. These data strongly suggest a fundamental issue with the taxonomic identity of the source material used for its assembly. Notably, this published genome was reportedly sampled from Yunnan Province (Feng et al., 2021). Meanwhile, two *A. formosana* specimens from Sichuan Province are archived at the Chengdu Institute of Biology, Chinese Academy of Sciences (https://www.cvh.ac.cn/spms/detail.php?id=d7e77a3a), underscoring the need to validate distribute localities for *A. formosana* (Liu and Sylvia, 2006; Lee et al., 2022). For *A. donax*, integrating the reassembled chloroplast genomes of *A. donax* cv. Lvzhou No.1 with the datasets used by Luo et al. (2024) revealed that it clustered with other *A. donax* chloroplast genomes, contradicting Luo et al.’s hypothesis that “*A. donax* cv. Lvzhou No.1 (OQ993163.1) may represent a variety with a different genetic origin from the other *A. donax*” (**Supplementary File S2**; **Supplementary Figure S8**; Luo et al., 2024). Crucially, clone 0004 exhibited distinct morphological traits (e.g., sprawling growth habit, taller culms) despite chloroplast genome identity with *A. donax* cv. Lvzhou No.1 (Ren et al., 2023b; Luo et al., 2024). Molecular clock analyses reveal synchronized divergence events between East Asian and Mediterranean lineages: *P. australis* populations in China’s Hexi Corridor diverged from European counterparts 0.186 Mya (Qiu and Cui, 2021), contemporaneous with the ~0.17 Mya split between *A. donax* lineages (**Figure 7**). This temporal synchronicity reinforces hypotheses of human-mediated dispersal facilitating Mediterranean *A. donax* expansion (Hardion et al., 2017b).

For the *A. plinii* complex, divergence time estimation indicates that *A. smaragdina* and *A. plinii* diverged approximately 2.29 MYA (**Figure 7**). This period, characterized by initial cooling and mild rainfall reduction followed by the consolidation of an arid Mediterranean climate, temporally coincides with both the divergence of *A. plinii* complex taxa and analogous speciation patterns observed in *Avena* species (Liu et al., 2017). Furthermore, the divergence among the three species within the *A. plinii* complex requires further clarification. Neither nuclear genome nor chloroplast genome analyses have clustered *A. plinii* BO and *A. plinii* RO into a single clade (**Figure 6**; Jike et al., 2020), likely attributable to both the slower evolutionary rate of the chloroplast genome and the limited nuclear gene dataset analyzed (comprising only 144 genes).

Regarding *A. micrantha* within this complex, Jike et al. (2020) proposed that it likely originated from hybridization between *A. plinii* and a low-ploidy fertile Asian *A. donax*. The identification of *A. smaragdina* provides evidence supporting an alternative hypothesis: *A. micrantha* more likely originated from hybridization between *A. plinii* and *A. smaragdina*. The supporting evidence is as follows (**Supplementary Table S7**): (1) Morphologically, *A. smaragdina* shares similarities with *A. micrantha* in rhizome growth patterns, exhibiting limited growth, which contrasts with the spreading growth habit observed in *A. donax*. Furthermore, the culm diameter under the panicle of *A. micrantha* (>5mm) is partially similar to that of *A. smaragdina* (4.7-12.9 mm), exceeding 5 mm. This characteristic also helps distinguish *A. micrantha* from *A. plinii* (< 4 mm) and *A. donaciformis* (< 4 *mm*). (2) The nearly identical pollen germination rates between *A. smaragdina* (12.7%) and *A. plinii* (7.8%-8%) suggest reproductive compatibility, which is a prerequisite for successful hybridization. In contrast, the pollen germination rate for *A. donax* is 0%. (3) Cytological analysis reveals that the chromosome numbers of both *A. smaragdina* and *A. micrantha* are approximately 72. This number falls within the range (72-114) reported for some *A. plinii* accessions, indicating a shared cytological background that could facilitate hybridization. (4) Phylogenetic reconstruction based on chloroplast genomes indicates a closer genetic relationship among *A. micrantha*, *A. plinii*, and *A. smaragdina* than with *A. donax*.

In conclusion, integrative analyses of morphological traits, chromosome numbers, MNP markers, and chloroplast genomes provide compelling evidence that *A. smaragdina* represents a distinct species within *Arundo*, diverging from *A. donax* and other species. At the nuclear genome level, *A. smaragdina* exhibits genetic divergence from *A. donax* based on MNP analysis, whereas at the chloroplast genome level, it shares structural similarities with the Chinese mainland *A. donax* and *A. plinii*. Morphologically, *A. smaragdina* combines high-yield characteristics of *A. donax* and *A. micrantha* with pollen germination traits similar to *A. plinii*. Collectively, these findings demonstrate that *A. smaragdina* occupies a pivotal evolutionary role within the genus *Arundo*. The discovery of this species provides a critical missing link for elucidating both the evolutionary history and biogeographic dispersal of the genus, thereby establishing a robust foundation for future initiatives in breeding programs, conservation strategies, and sustainable utilization of its members.

## Data Availability

The raw sequence data reported in this paper have been deposited in the Genome Sequence Archive (Genomics, Proteomics & Bioinformatics 2021) in National Genomics Data Center (Nucleic Acids Res 2022), China National Center for Bioinformation/Beijing Institute of Genomics, Chinese Academy of Sciences (GSA: CRA027255) that are publicly accessible at https://ngdc.cncb.ac.cn/gsa.
