# Supplementary material for: Arundo smaragdina (Poaceae): a novel species revealed by integrative taxonomy and its implications for the phylogeny of the genus
Source: Front Plant Sci. 2025 Nov 17;16:1660442. doi: 10.3389/fpls.2025.1660442 (PMC12666694; doi:10.3389/fpls.2025.1660442)
Supplement: Supplementary file 1 [file Supplementaryfile1.zip › Supplementary File 1/Table 1.DOCX]

Supplementary Material

# Supplementary Data

**Supplementary File 1.** Sequence variation plots among the *Arundo* chloroplast genomes. Annotated genes are displayed on the top. The color legend is summarized in the lower left-hand corner. “*” indicates that the genome was assembled from transcriptome data. Notably, the reference genome *A. formosana* (NC_054211.1) displayed significant divergence from *A. donax* (NC_037077.1) across multiple regions, particularly in non-coding regions spanning 3-9 k bp, 12-1 k bp, 43-49 and 50-80 k bp, where multiple segmented regions with sequence similarity below 60% were observed. In contrast, the near-complete chloroplast genome of *A. formosana* assembled from transcriptome data exhibited higher overall similarity to *A. donax*. For instance, in the 50-100 k bp region, the near-complete chloroplast genome of *A. formosana* was more similar to *A. donax* than the reference genome *A. formosana* (NC_054211.1) was to *A. donax*.

# Supplementary Figures and Tables

## Supplementary Figures


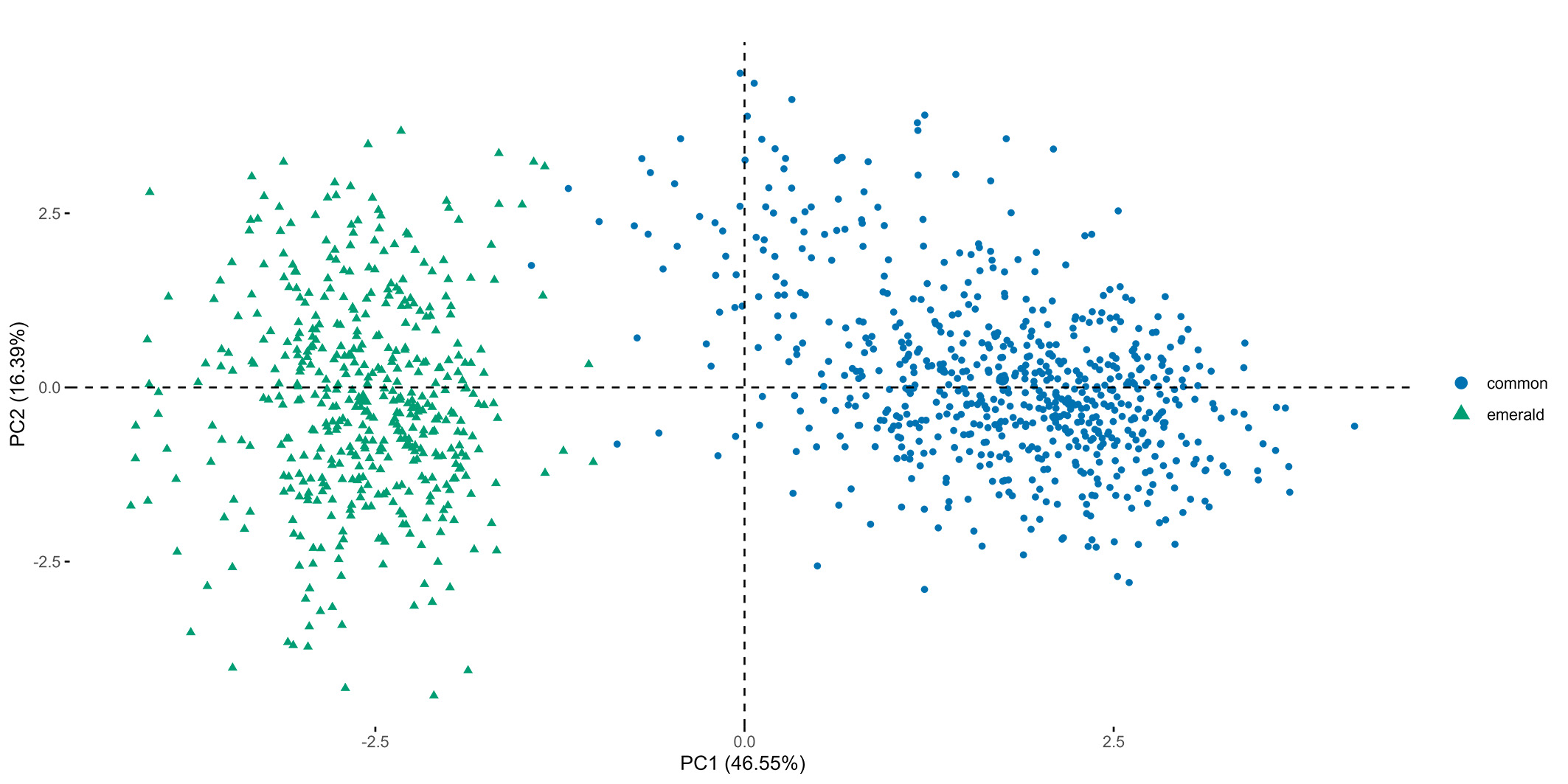


**Supplementary Figure 1.** Principal component analysis (PCA) of 118 clones based on 11 morphological traits.


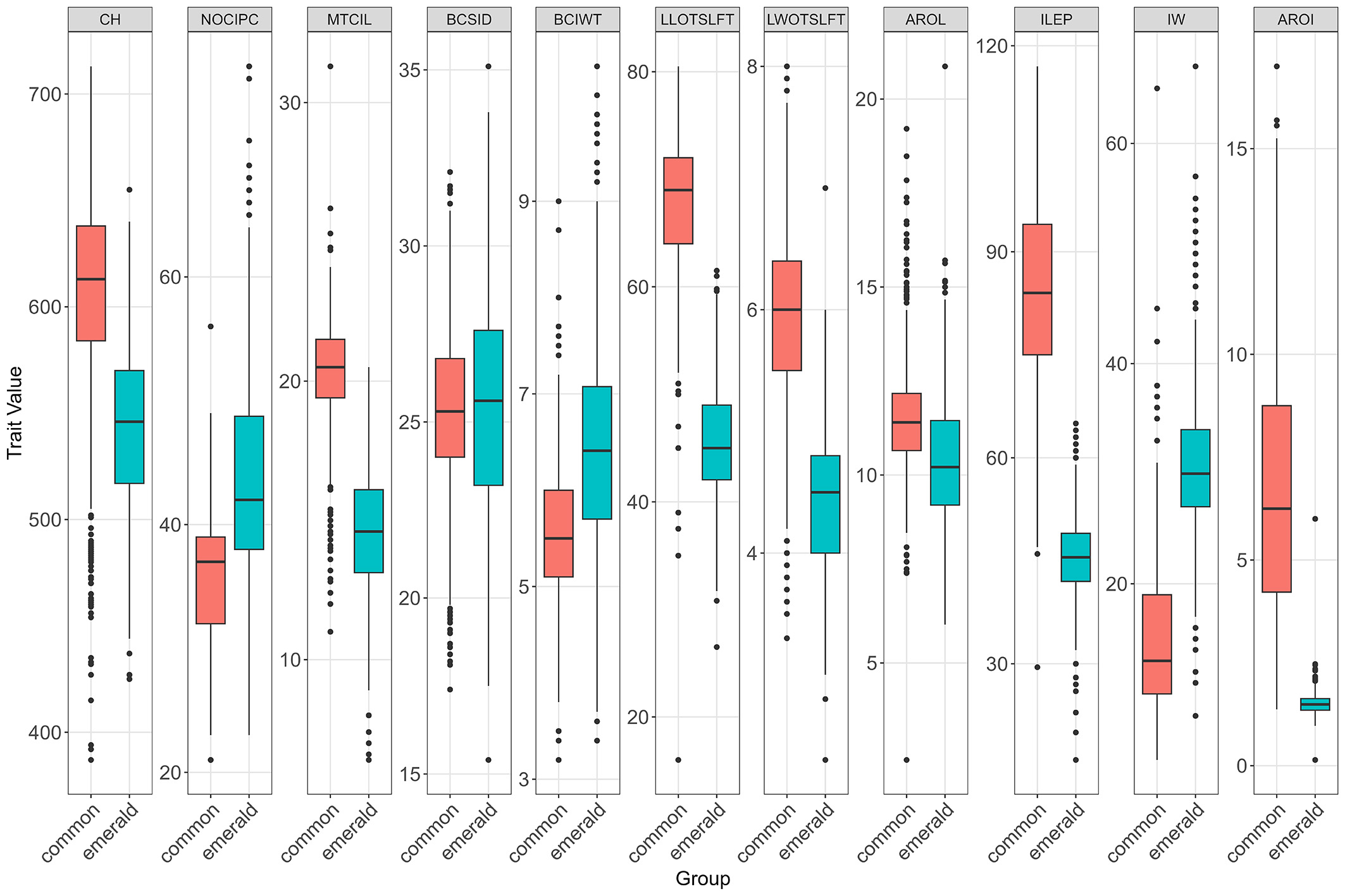


**Supplementary Figure 2.** Box-and-whiskers plots of 11 morphological traits. Traits include: CH: Culm height (cm); NOCIPC: Number of culm internodes per culm; MTCIL: Middle ten culm internodes length (cm); BCSID: Basal culm stem internode diameter (mm); BCIWT: Basal culm internode wall thickness (mm); LLOTSLFT: Leaf length of the sixth leaf from top (cm); LWOTSLFT: Leaf width of the sixth leaf from top; AROL: Aspect ratio of leaf (LLOTSLFT/LWOTSLFT); IW: Inflorescence width (cm); ILEP: Inflorescence length (excluding peduncle) (cm); AROI: Aspect ratio of inflorescence (ILEP/IW).


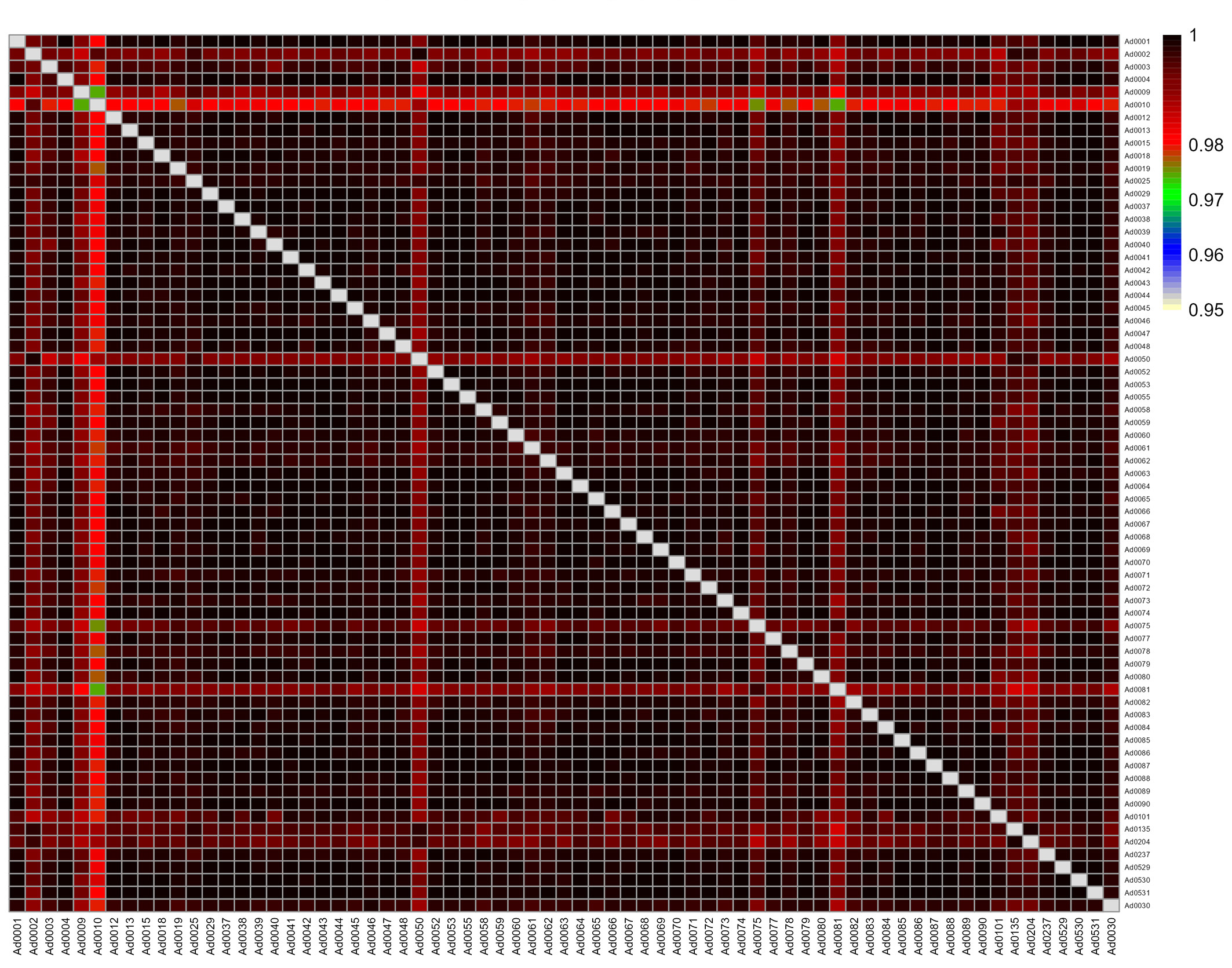


**Supplementary Figure 3.** Heatmap of genetic similarity among clones of the common group.


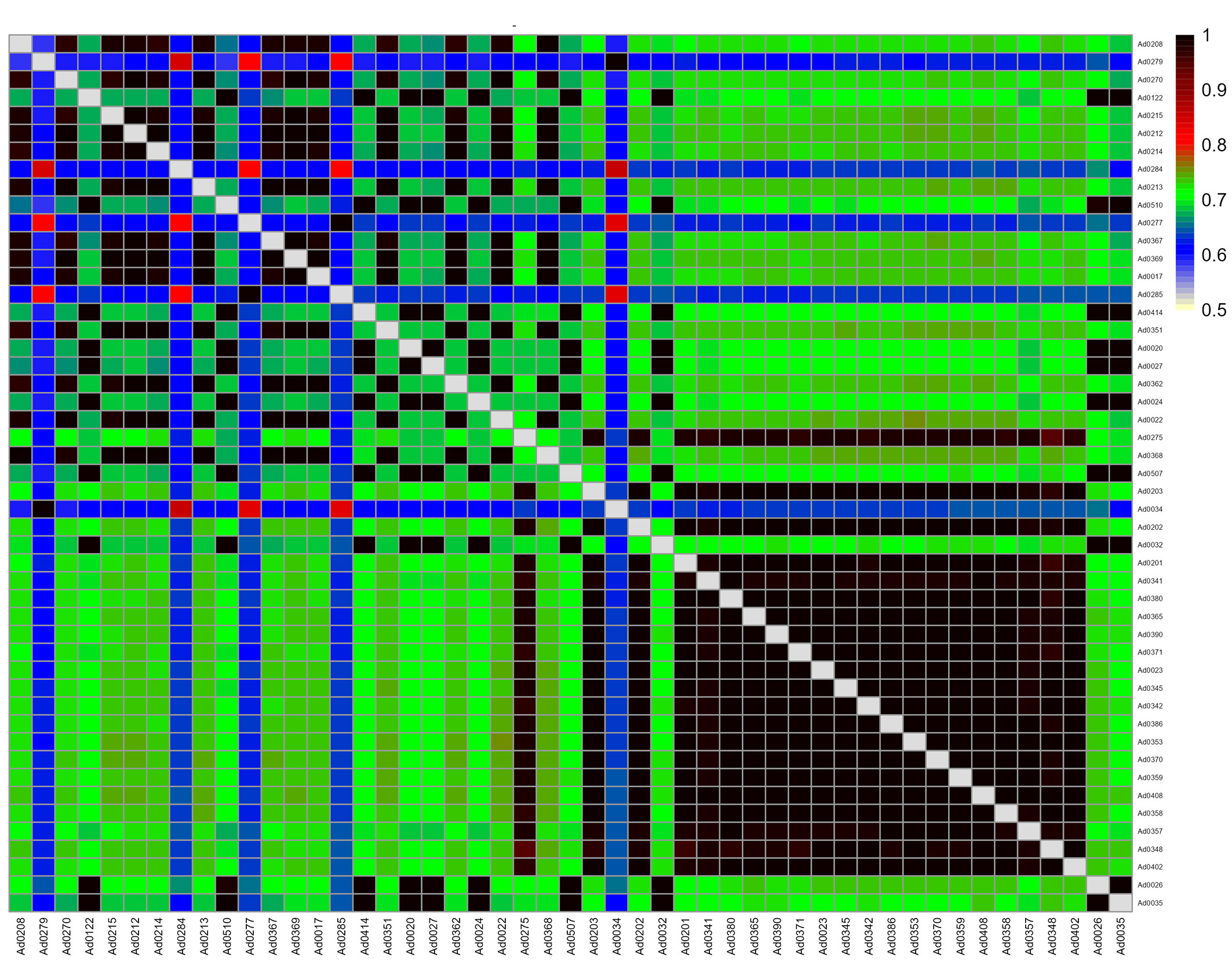


**Supplementary Figure 4.** Heatmap of genetic similarity among clones of the emerald group.


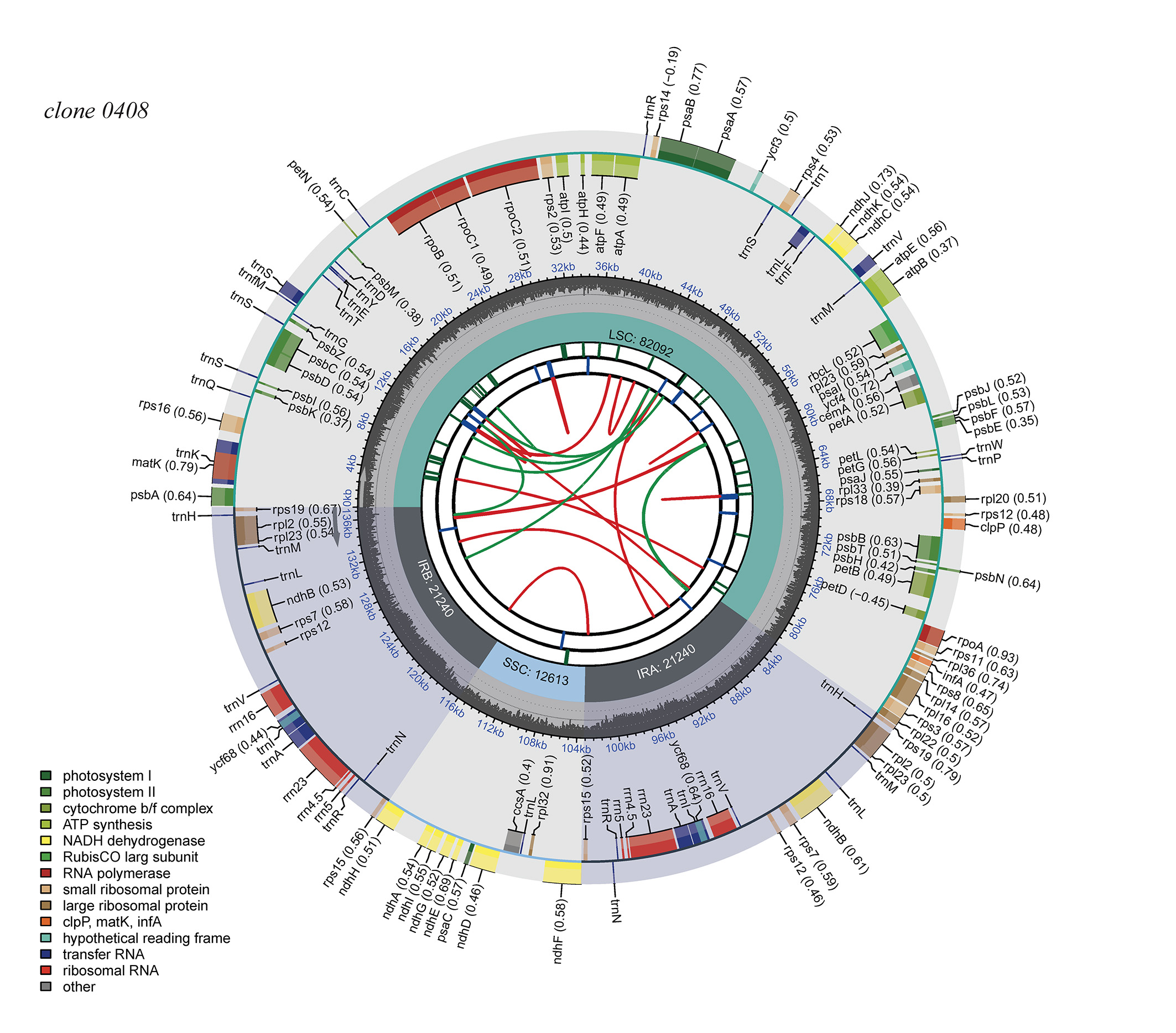


**Supplementary Figure 5.** Gene map of the clone 0408 chloroplast genome. From the center outward, the first track shows the dispersed repeats. The dispersed repeats consist of direct (D) and Palindromic (P) repeats, connected with red and green arcs. The second track shows the long tandem repeats as short blue bars. The third track shows the short tandem repeats or microsatellite sequences as short bars with different colors. The colors, the type of repeat they represent, and the description of the repeat types are as follows. Black: c (complex repeat); Green: p1 (repeat unit size = 1); Yellow: p2 (repeat unit size = 2); Purple: p3 (repeat unit size = 3); Blue: p4 (repeat unit size = 4); Orange: p5 (repeat unit size = 5); Red: p6 (repeat unit size = 6). The small single-copy (SSC), inverted repeat (IRa and IRb), and large single-copy (LSC) regions are shown on the fourth track. The GC content along the genome is plotted on the fifth track. The genes are shown on the sixth track. The optional codon usage bias is displayed in the parenthesis after the gene name. Genes are color-coded by their functional classification. The transcription directions for the inner and outer genes are clockwise and anticlockwise, respectively. The functional classification of the genes is shown in the bottom left corner.


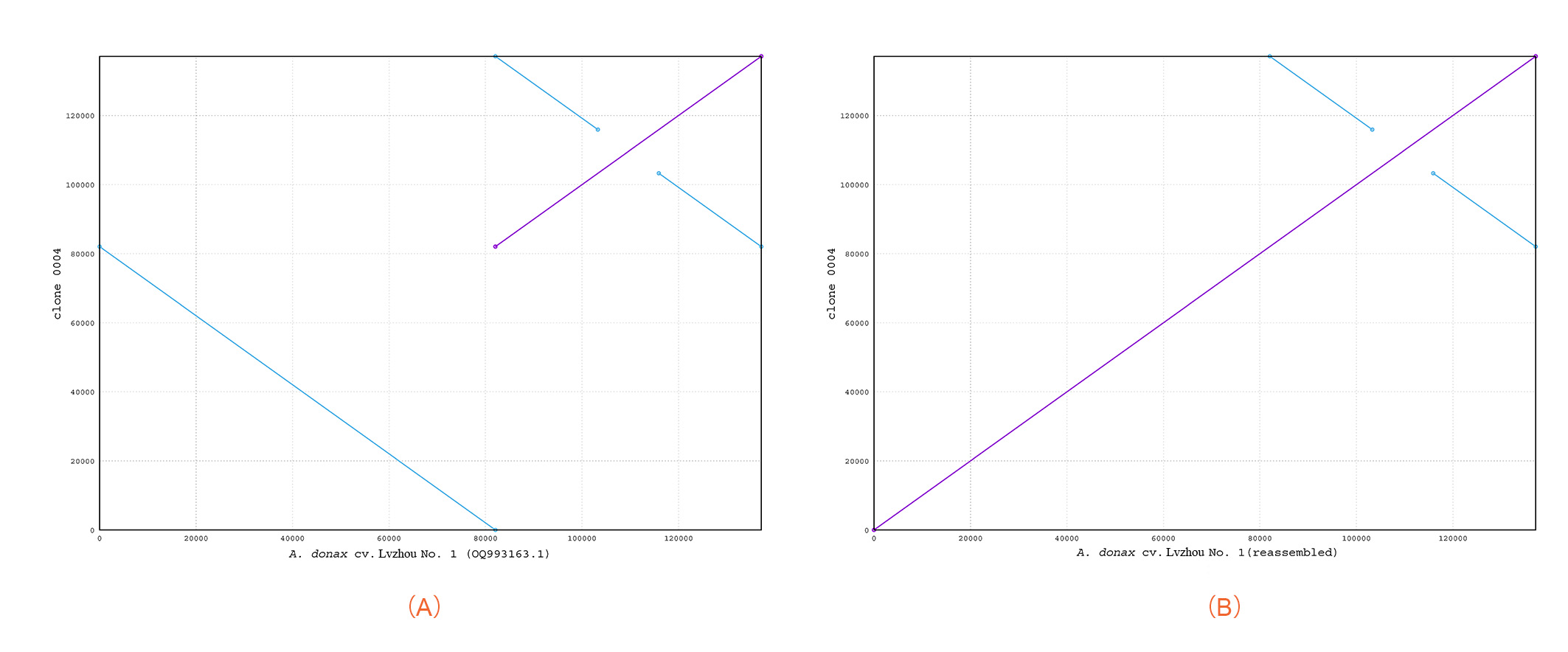


**Supplementary Figure 6.** Chloroplast genome comparison between *A. donax* cv. Lvzhou No. 1 and clone 0004 using the nucmer package (default parameters).


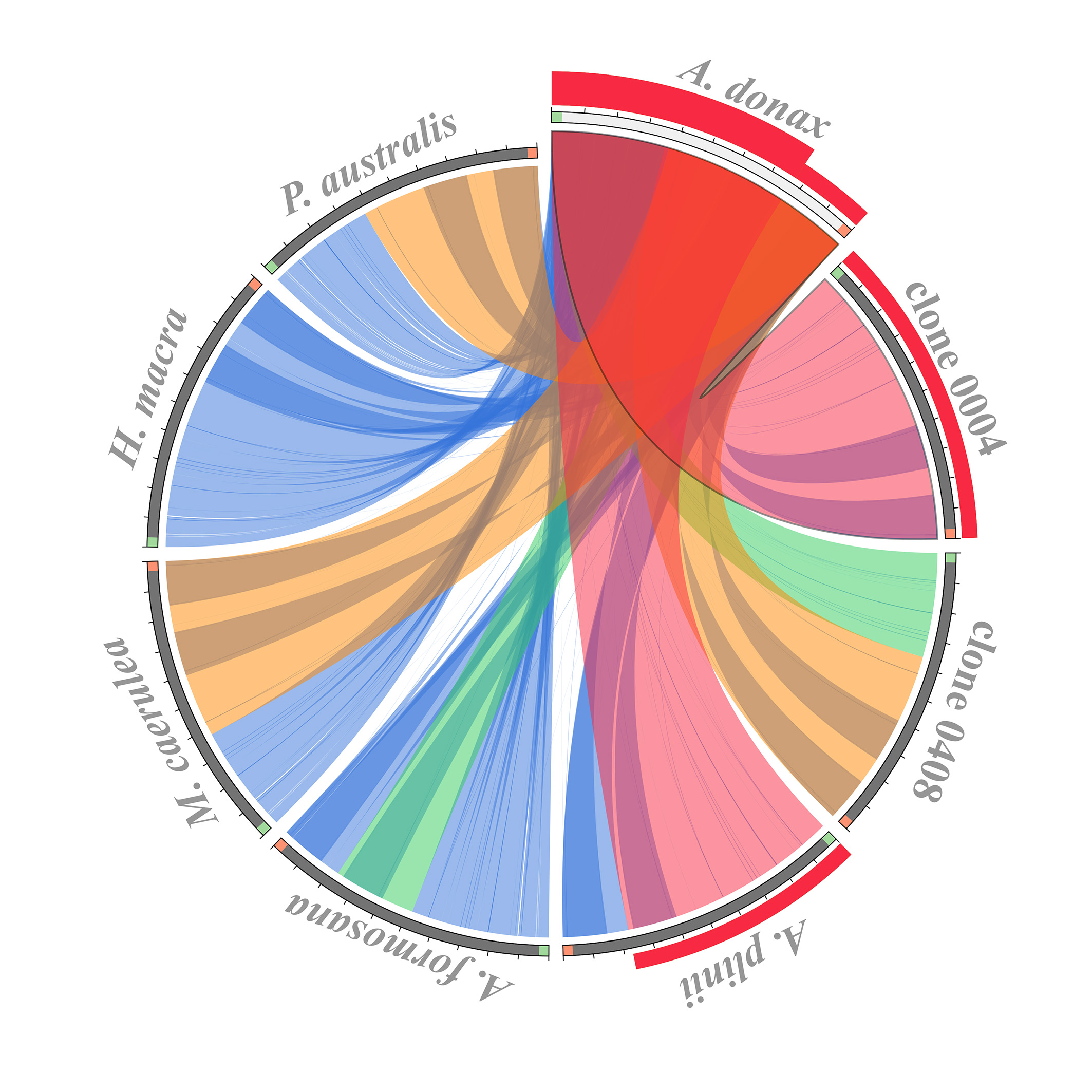


**Supplementary Figure 7.** Collinear analysis of *A.donax* and its related species. The ribbons ('score/max' ratio) showed the homologous sequences between species, with blue<=0.25, green<=0.50, orange<=0.75, red>0.75. It is evident that the reference genome of *A. formosana* (NC_054211.1) exhibits less than 50% similarity to *A. donax*, and even lower similarity compared to *P. australis*, *H. macra*, or *M. caerulea*.


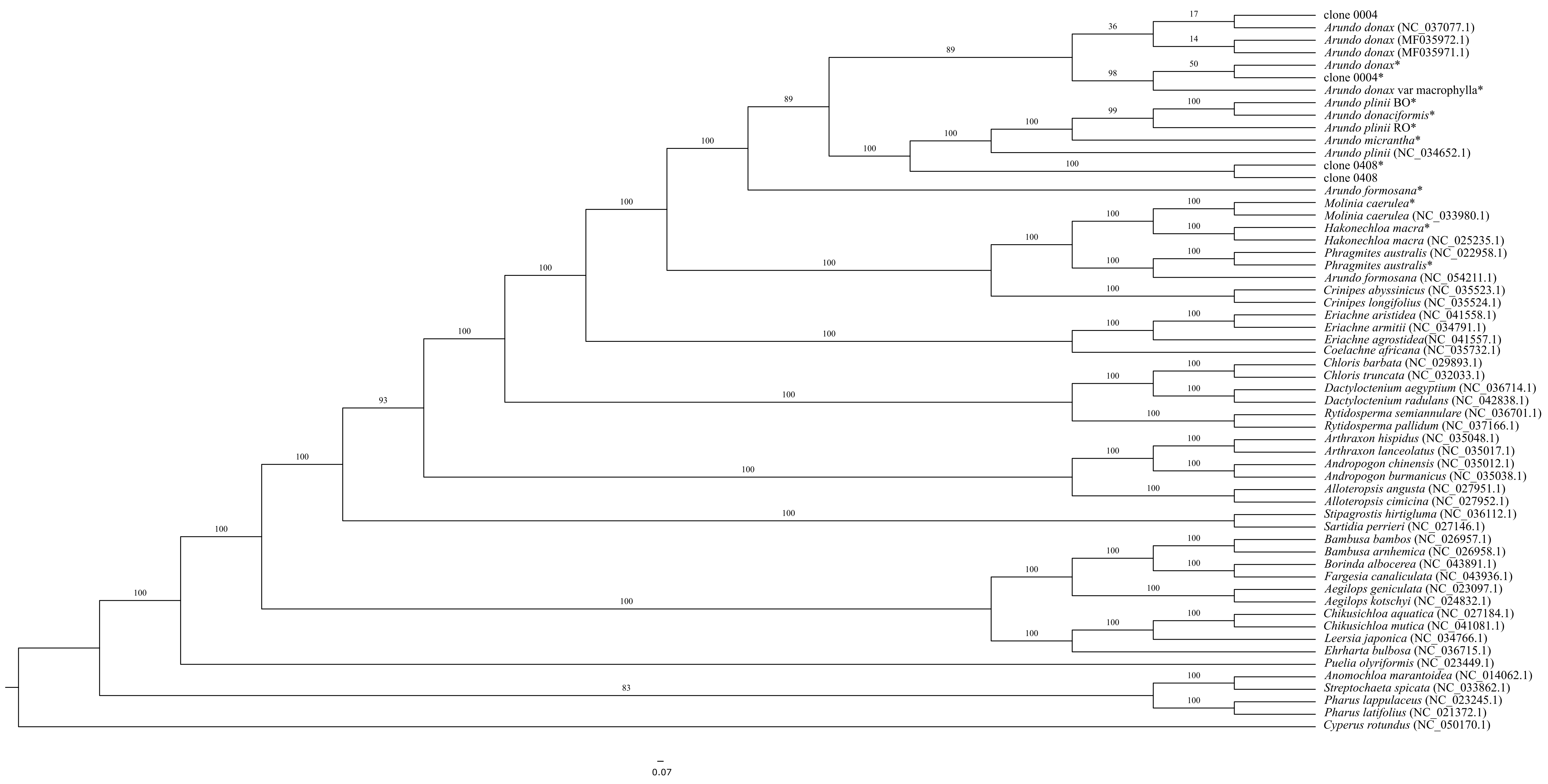


**Supplementary Figure 8.** Phylogenetic tree containing chloroplast genome sequence of 57 grasses was constructed by maximum likelihood (ML) strategy using IQ-TREE. “*” indicates that the genome was assembled from transcriptome data. Interestingly, the published *A. formosana* (NC_054211.1) chloroplast genome did not cluster with the near-complete chloroplast genome, but instead grouped with *P. australis*. Notably, this published genome was reportedly sampled from Yunnan Province. Meanwhile, two *A. formosana* specimens from Sichuan Province are archived at the Chengdu Institute of Biology, Chinese Academy of Sciences (https://www.cvh.ac.cn/spms/detail.php?id=d7e77a3a), underscoring the need to validate distribute localities for *A. formosana*.


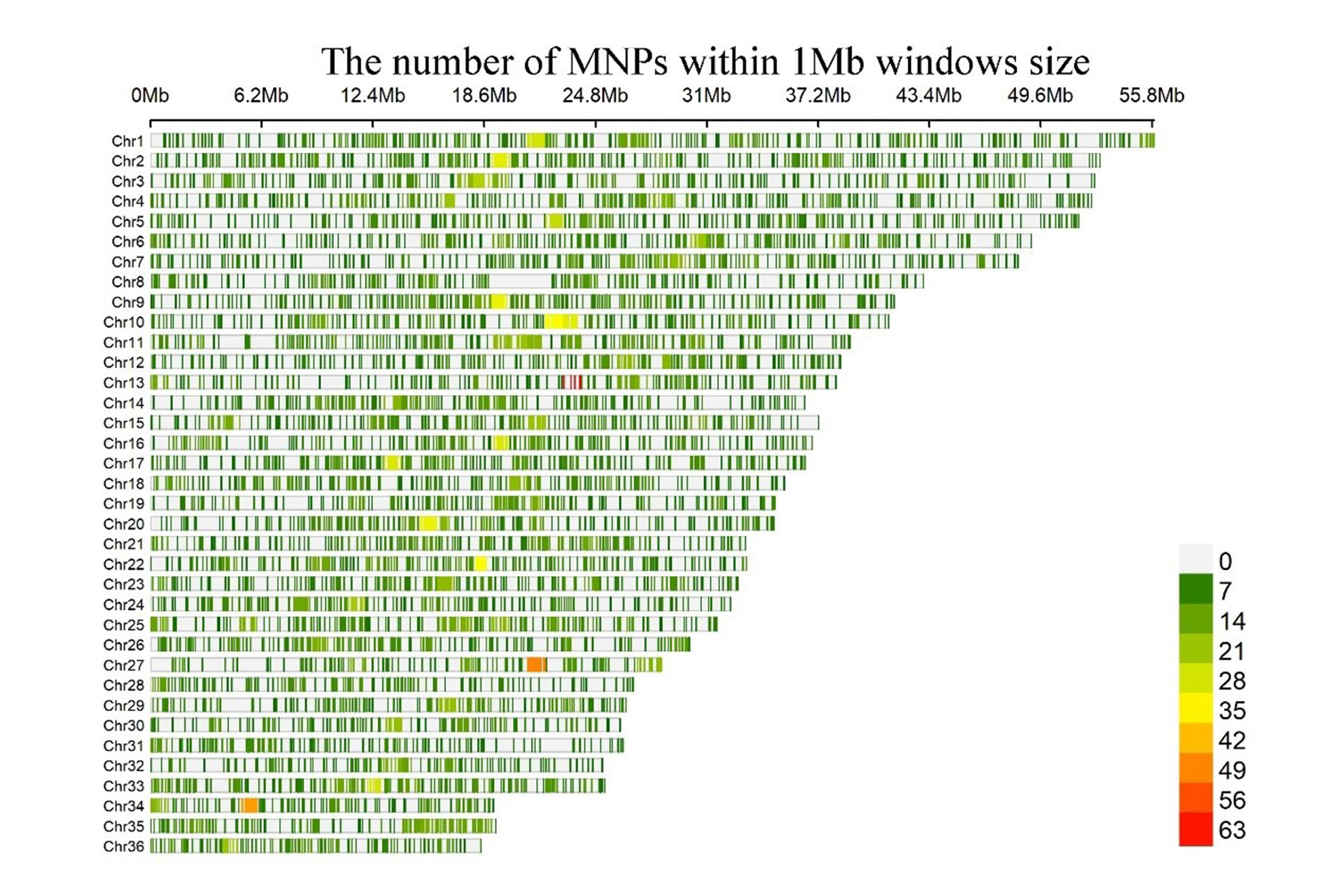


**Supplementary Figure 9.** Chromosomal distribution of 1,100 MNP markers in *A. donax*.

## Supplementary Tables

**Supplementary Table 1.** Trait variation statistics for 118 clones

**Supplementary Table 2.** The PCA of 11 phenotypic traits among 118 clones

**Supplementary Table 3.** Genetic similarity between 118 clones based on 1100 MNP markers

**Supplementary Table 4.** Comparison of Chloroplast Genomes

**Supplementary Table 5.** The comparison of two chloroplast genome sequences of *A. formosana*

**Supplementary Table 6.** Sequence comparison of the IR regions

**Supplementary Table 7.** Comparison of species traits in the genus *Arundo*
