# Supplementary figures and images for: Arundo smaragdina (Poaceae): a novel species revealed by integrative taxonomy and its implications for the phylogeny of the genus
Source: Front Plant Sci. 2025 Nov 17;16:1660442. doi: 10.3389/fpls.2025.1660442 (PMC12666694; doi:10.3389/fpls.2025.1660442)

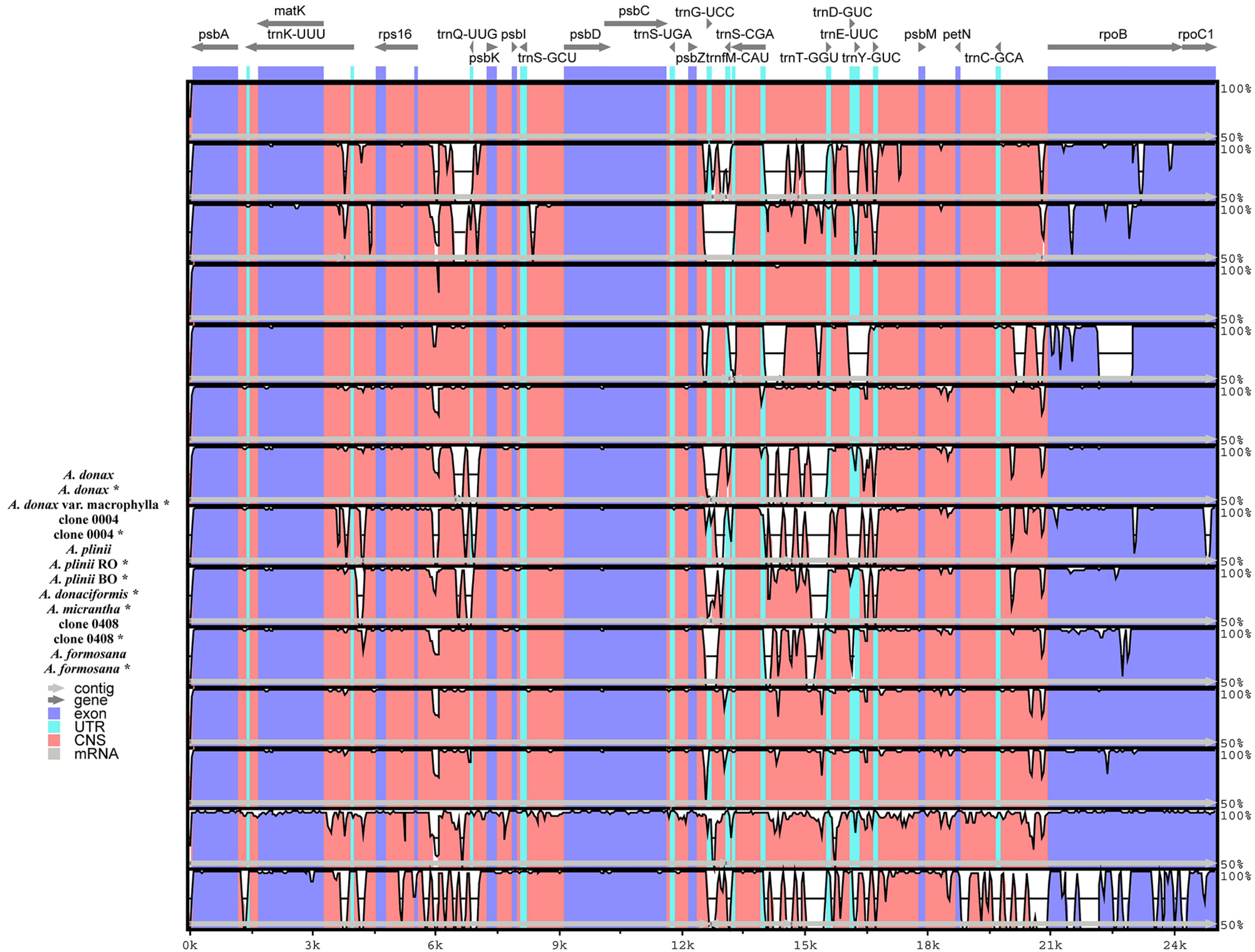

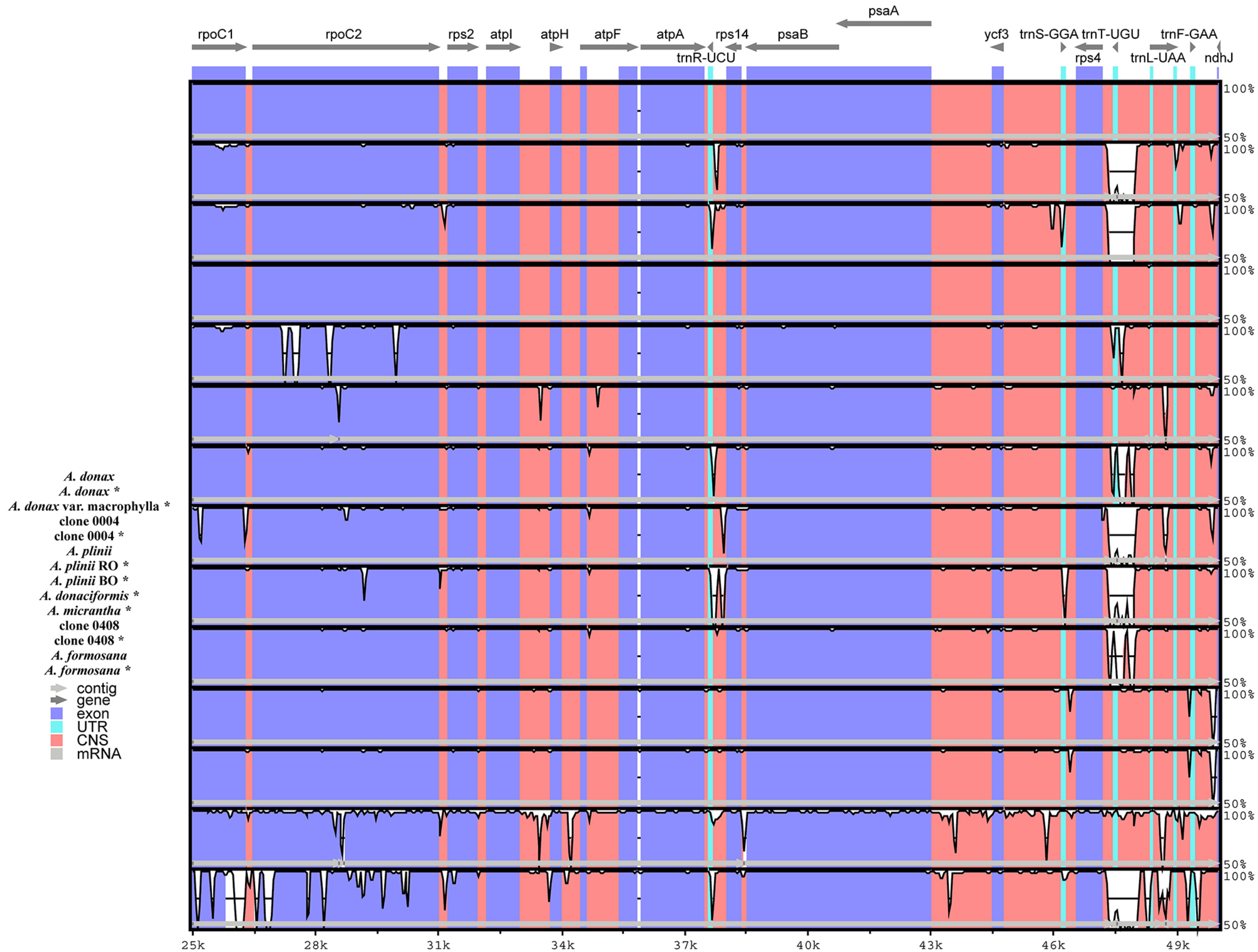

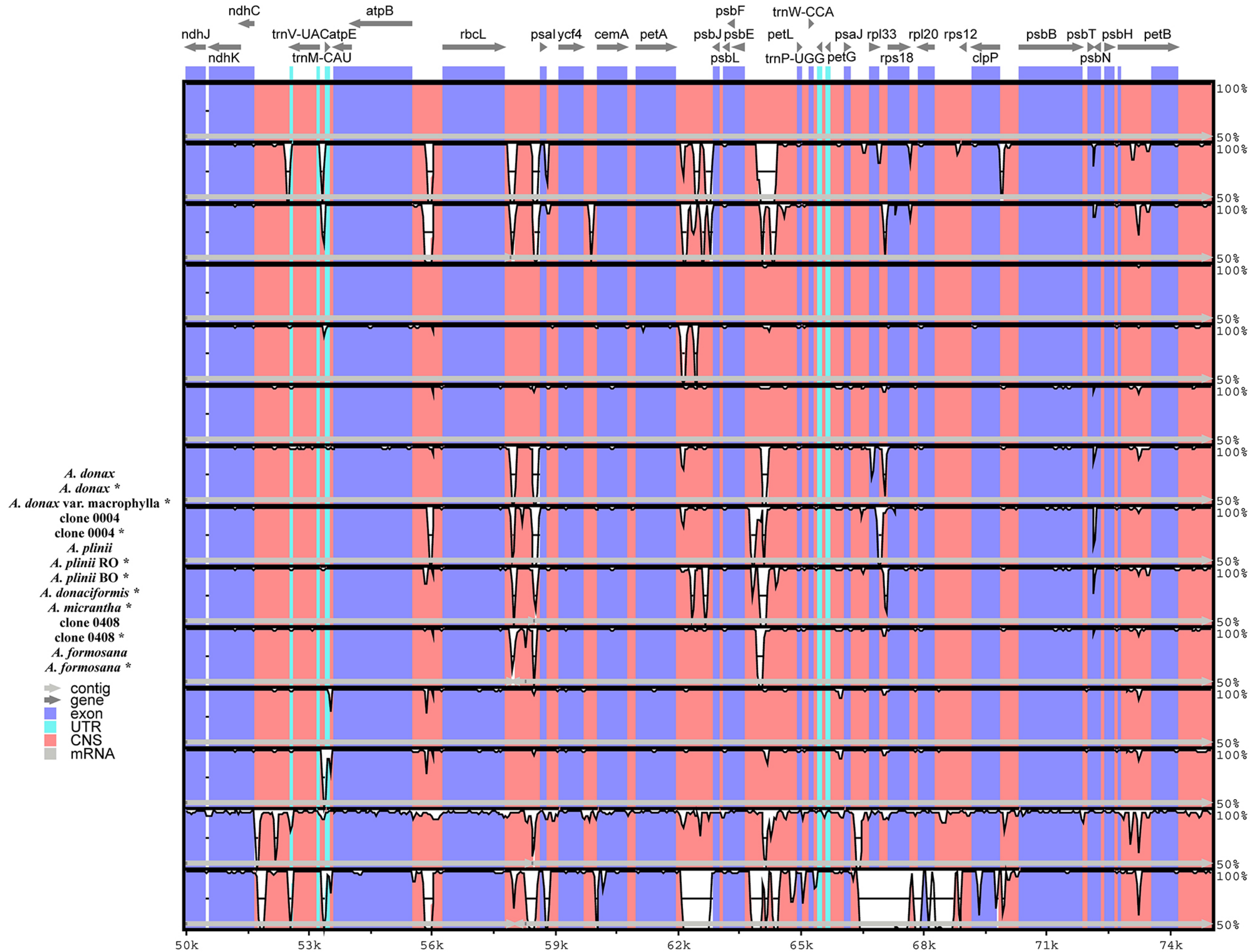

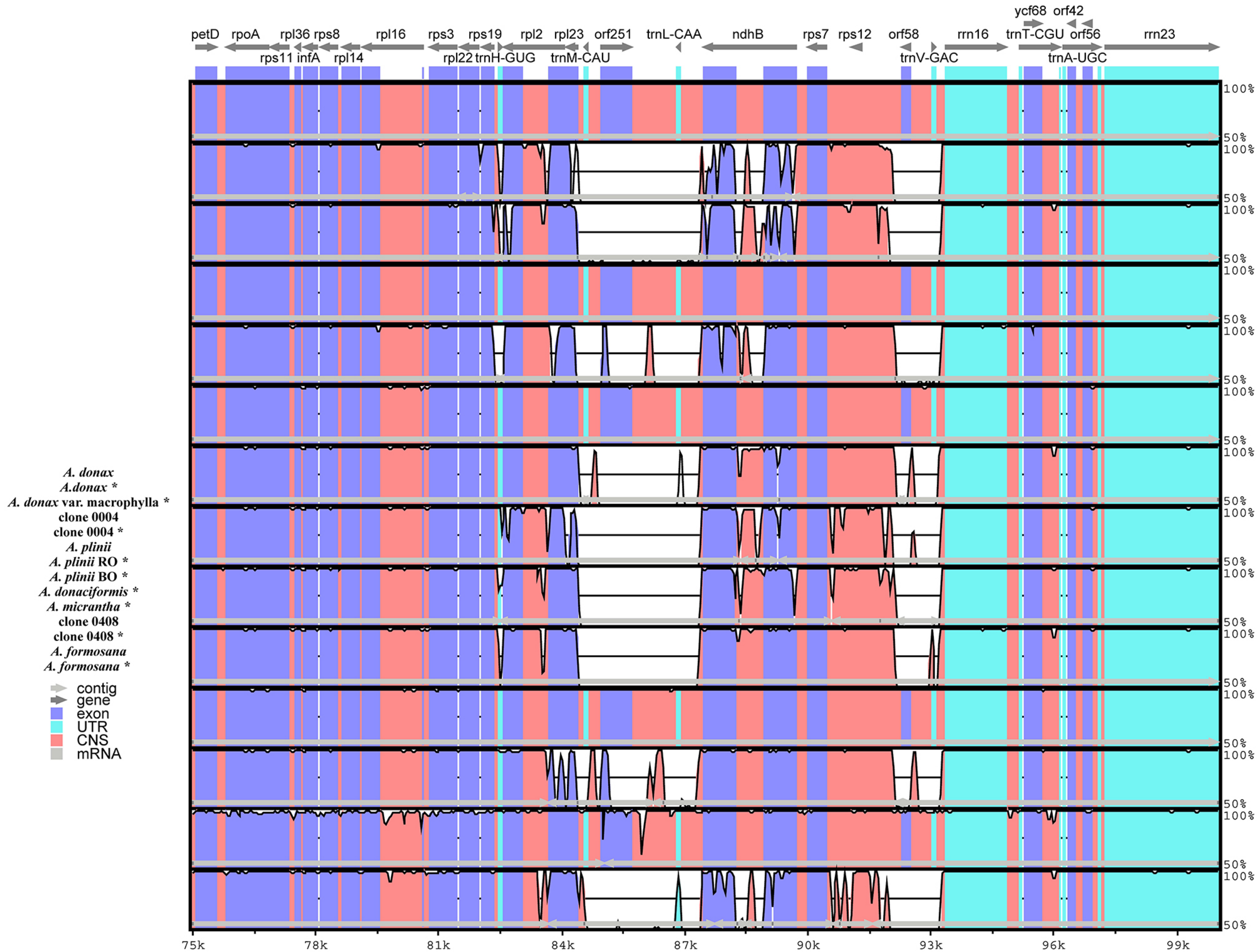

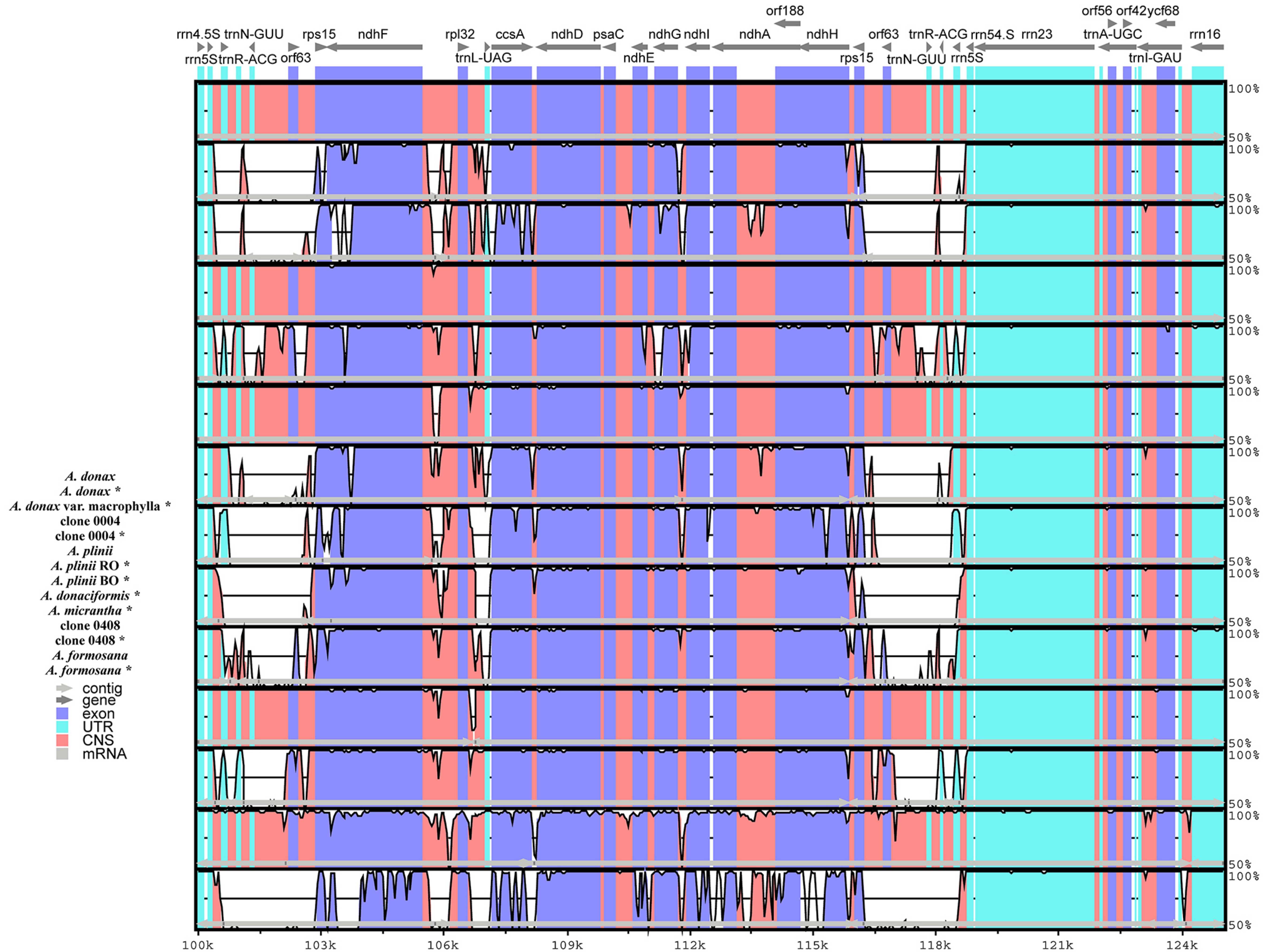

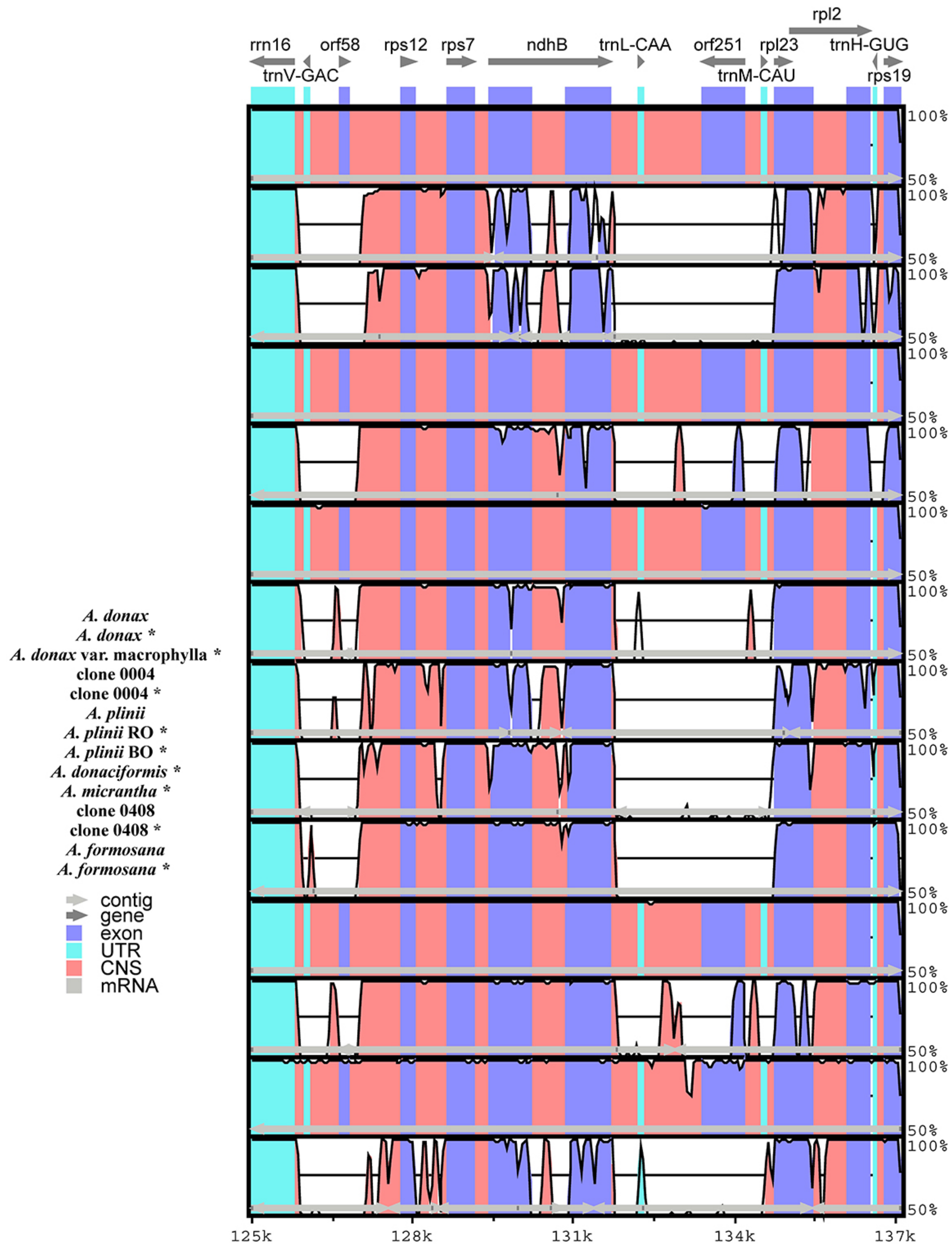

Supplement: Supplementary file 1 [file Supplementaryfile1.zip › Supplementary File 1/Data Sheet 1.PDF]
